# Comparative Transcriptional and Translational Analysis of Leptospiral Outer Membrane Protein Expression in Response to Temperature

**DOI:** 10.1371/journal.pntd.0000560

**Published:** 2009-12-08

**Authors:** Miranda Lo, Stuart J. Cordwell, Dieter M. Bulach, Ben Adler

**Affiliations:** 1 Australian Research Council Centre of Excellence in Structural and Functional Microbial Genomics, Department of Microbiology, Monash University, Melbourne, Australia; 2 School of Molecular and Microbial Biosciences, University of Sydney, Sydney, Australia; 3 Victorian Bioinformatics Consortium, Monash University, Melbourne. Australia; Institut Pasteur, France

## Abstract

**Background:**

Leptospirosis is a global zoonosis affecting millions of people annually. Transcriptional changes in response to temperature were previously investigated using microarrays to identify genes potentially expressed upon host entry. Past studies found that various leptospiral outer membrane proteins are differentially expressed at different temperatures. However, our microarray studies highlighted a divergence between protein abundance and transcript levels for some proteins. Given the abundance of post-transcriptional expression control mechanisms, this finding highlighted the importance of global protein analysis systems.

**Methodology/Principal Findings:**

To complement our previous transcription study, we evaluated differences in the proteins of the leptospiral outer membrane fraction in response to temperature upshift. Outer membrane protein-enriched fractions from *Leptospira interrogans* grown at 30°C or overnight upshift to 37°C were isolated and the relative abundance of each protein was determined by iTRAQ analysis coupled with two-dimensional liquid chromatography and tandem mass spectrometry (2-DLC/MS-MS). We identified 1026 proteins with 99% confidence; 27 and 66 were present at elevated and reduced abundance respectively. Protein abundance changes were compared with transcriptional differences determined from the microarray studies. While there was some correlation between the microarray and iTRAQ data, a subset of genes that showed no differential expression by microarray was found to encode temperature-regulated proteins. This set of genes is of particular interest as it is likely that regulation of their expression occurs post-transcriptionally, providing an opportunity to develop hypotheses about the molecular dynamics of the outer membrane of *Leptospira* in response to changing environments.

**Conclusions/Significance:**

This is the first study to compare transcriptional and translational responses to temperature shift in *L. interrogans*. The results thus provide an insight into the mechanisms used by *L. interrogans* to adapt to conditions encountered in the host and to cause disease. Our results suggest down-regulation of protein expression in response to temperature, and decreased expression of outer membrane proteins may facilitate minimal interaction with host immune mechanisms.

## Author Summary

Leptospirosis, caused by *Leptospira* spp., is a disease of worldwide significance affecting millions of people annually. Bacteria of this species are spread by various carrier animals, including rodents and domestic livestock, which shed the leptospires via their urine into the environment. Humans become infected through direct contact with carrier animals or indirectly via contaminated water or soil. Temperature is a key trigger used by many bacteria to sense changes in environmental conditions, including entry from the environment into the host. This study was the first comprehensive research into changes occurring in the outer membrane of *Leptospira* in response to temperature and how these changes correlate with gene expression changes. An understanding of the regulation and function of these proteins is important as they may provide an adaptation and survival advantage for the microorganism which may enhance its ability to infect hosts and cause disease. Our data suggest regulation of proteins in the outer membrane which may possibly be a mechanism to minimise interactions with the host immune response.

## Introduction

Leptospirosis is a widespread zoonotic disease caused by spirochetes of the genus *Leptospira*, which can persist for prolonged periods in the environment and cause human infection via contact with infected animals or contaminated soil/water [1]. The organism has a broad host range and maintenance hosts harbor pathogenic *Leptospira* spp. in their proximal renal tubules, commonly resulting in shedding of bacteria in the urine and subsequently the environment, thereby providing a potential source of infection. An exception to this cycle is *L. borgpetersenii* serovar Hardjo which does not survive well in the environment and generally appears to require direct host-to-host transmission, likely due to its smaller genome and loss of genes required for environmental survival [2]. In tropical countries, large outbreaks of human leptospirosis have occurred following severe floods, while in developed countries cases usually occur through occupational contact or recreational activities [3],[4]. Human leptospirosis is extremely variable in its clinical manifestations, ranging from mild flu-like symptoms through to rapidly fatal forms involving multiple organ failure, with death occurring in 5–25% of severe cases [5],[6]. Currently, little is known about pathogenesis mechanisms or transcriptional regulation in *Leptospira* spp.

Temperature is an environmental factor known to affect leptospiral protein expression, and is a key trigger used by many bacteria to sense changes in environmental conditions, including entry from the environment into the host. *Leptospira* spp. can grow in artificial media at a range of temperatures that reflect the conditions found in the environment and the mammalian host. Therefore, we had previously investigated transcriptional changes between cultures grown at 20°C, 30°C, 37°C or 39°C reflecting ambient temperatures in the environment, growth under laboratory conditions (*Leptospira* spp. are routinely cultured at 30°C), and temperatures in healthy and febrile hosts respectively [7]. Additionally, cultures grown at 30°C then shifted overnight to 37°C were compared with those grown long-term at 30°C and 37°C to identify genes potentially expressed in the early stages of infection or during transition from the environment into the host. Comparison of data sets provided novel insights into possible transcriptional changes at different stages of infection. However, our microarray data did not correlate completely with findings from previous studies which showed that expression of some proteins is temperature-regulated, namely LipL36 [8],[9] and Qlp42 (or LipL45) [10]. Given the abundance of post-transcriptional expression control mechanisms, for example, translational regulation by small RNAs [11],[12], this finding highlighted the importance of global protein analysis systems.

Proteomic advances such as the development of multidimensional protein identification technology (MudPIT) [13],[14] and isobaric tags such as the iTRAQ reagents [15] have enabled large scale identification and determination of relative protein abundance between different samples simultaneously. The technology has been applied to studying global proteomic changes in response to environmental cues in various prokaryotic organisms such as *Escherichia coli*, *Methanosarcina acetivorans*, *Desulfovibrio vulgaris*, and *Nostoc* sp. [16]–[20].

Expression of various leptospiral outer membrane proteins has previously been shown to be temperature-regulated. For example, LipL45 and Hsp15 were up-regulated [10],[21] while LipL36 was down-regulated with temperature increase [8],[22]. Therefore, to complement the transcription study, we evaluated the changes in the protein constituents of the leptospiral outer membrane fraction in response to temperature upshift, as proteins which are up-regulated in response to temperature may be important upon host entry and establishing infection. To our knowledge, the study presented here is the first to compare transcriptional and translational responses to temperature shift in *L. interrogans*.

## Materials and Methods

### Culture conditions

*L. interrogans* serovar Lai was grown in EMJH medium [23] at 30°C until mid-log phase of growth (<5×10^8^ cells/ml) before harvesting for outer membrane extraction. For the overnight upshift to 37°C, cultures were grown to 2.5×10^8^ cells/ml at 30°C then incubated at 37°C for 16–20 h before harvesting. Cell count was determined as described previously [24]. At the time of harvest, the cell 30°C and 37°C upshift cultures were enumerated at 3.4×10^8^ and 3.6×10^8^ cells/ml respectively.

### Extraction of leptospiral outer membranes

*L. interrogans* outer membrane samples were prepared by Triton X-114 extraction as described previously [25],. Briefly, leptospires were washed three times in phosphate-buffered saline-5 mM MgCl_2_ by centrifugation at 9,000×*g* for 10 min. Outer membrane material was then extracted using 1% protein-grade Triton X-114 (Calbiochem) in 150 mM NaCl-10 mM Tris (pH 8)-1 mM EDTA at 4°C. Insoluble material was removed by centrifugation at 17,000×*g* for 10 min, then CaCl_2_ to a final concentration of 20 mM was added to the supernatant which was subsequently passed through a 0.22 µm filter. Phase separation was achieved by warming the supernatant to 37°C, which was then subjected to centrifugation at 1,000×*g* for 10 min. The detergent phase was collected and proteins were purified by methanol/chloroform extraction. TX-114 preparations were checked for enrichment of OMPs and minimal contamination with inner membrane or cytoplasmic proteins by western immunoblot using antisera against OM, inner membrane and cytoplasmic markers (anti-LipL48, anti-ImpL63 and anti-GroEL respectively).

### Methanol/chloroform extraction of proteins

Detergent was removed from the protein samples by methanol/chloroform extraction as described previously [27]. To 0.1 ml of protein sample, 0.4 ml of methanol was added. The sample was then mixed and centrifuged at 9000×*g* for 10 s before adding another 0.1 ml of chloroform. The sample was again mixed, centrifuged at 9000×*g* for 10 s then 0.3 ml of water was added. The sample was again mixed vigorously and centrifuged at 9000×*g* for 1 min then the upper phase was removed. This was repeated twice before 0.3 ml of methanol was added to the remaining lower phase and the interphase containing precipitated proteins. After mixing, the sample was centrifuged at 9000×*g* for 2 min to pellet the protein which was then dried. Water was then added to each pellet to make a suspension and 10 µl was removed for determination of protein concentration using the bichinchoninic acid (BCA) protein assay according to the manufacturer's instructions (Pierce). The volume of sample containing 100 µg of each protein mixture was then taken from the suspension, dried and subjected to iTRAQ analysis.

### Isobaric peptide (iTRAQ) labeling

For each sample, 100 µg of protein were dissolved in 75 µl of 200mM triethylammonium bicarbonate buffer (TEAB) at pH 8.0 and 25 µl of 2% SDS, sonicated, reduced, blocked, digested, then labeled with the isobaric iTRAQ reagents as per the manufacturer's instructions (Applied Biosystems). For quantifying the effects of temperature upshift on abundance of outer membrane proteins from *L. interrogans* serovar Lai, two replicates of the 30°C and 37°C upshift samples were compared in the one experiment; the 30°C samples were labeled with tag_114_ and tag_115_ while the 37°C upshift samples were labeled with tag_116_ and tag_117_.

### Strong-cation exchange liquid chromatography (HPLC)

An Agilent 1100 quaternary HPLC pump (Agilent) with a PolyLC PolySulfoethyl A pre-packed column with a 5 µm particle size and column dimension of 200×2.1mm, 200 Å pore size was used for strong cation exchange chromatography (SCX). Buffer A was a solution with 5 mM KH_2_PO_4_ and 25% acetonitrile, pH 2.7 and buffer B was 5 mM KH_2_PO_4_ 350 mM KCl 25% acetonitrile, pH 2.7. The dried iTRAQ labeled samples were resuspended in buffer A and loaded onto the column. After sample loading and equilibrating with buffer A, buffer B concentration was increased to 10% then 10% to 45% over 70 minutes and finally increased to 100% and held at 100% for 10 minutes with a flow rate of 300 µl/min. The eluent of SCX was collected every 2 min at the beginning of the gradient and at 4 min intervals later.

### Reverse phase liquid chromatography/tandem MS (LC/MS-MS)

An Agilent 1100 nanoLC system (Agilent) coupled to an Applied Biosystems QSTAR XL mass spectrometer was used for separation and identification of peptides. The SCX fractions were resuspended in 100 µl of loading/desalting solution (0.1% trifluoroacetic acid and 2% acetonitrile 97.9% water) of which 39 µl were loaded on a reverse phase peptide Captrap (Michrom Bioresources) and desalted with the desalting solution at 10 µl/min for 13 min. After desalting, the trap was switched on line with a 150 µm×10 cm C_18_ 3 µm 300 Å ProteCol column (SGE). The buffer B (90% acetonitrile, 0.1% formic acid) concentration was increased from 5% to 90% over 120 min in three linear gradient steps to elute peptides. After peptide elution, the column was cleaned with 100% buffer B for 15 min and then equilibrated with buffer A (0.1% formic acid) for 30 min before the next sample injection. The reverse phase nanoLC eluent was subject to positive ion nanoflow electrospray analysis in an information dependant acquisition mode (IDA). In IDA mode a TOF-MS survey scan was acquired (m/z 370–1600, 0.5 s), with the three most intense multiply charged ions (counts >70) in the survey scan sequentially subjected to MS/MS analysis. MS/MS spectra were accumulated for 2 s in the mass range m/z 100–1600.

### Tandem mass spectra analysis and protein identification

The experimental nanoLC ESI MS/MS data were submitted to ProteinPilot (Applied Biosystems, trial version 1.0) for data processing. To identify the proteins present, the results were matched with our revised annotation of the *L. interrogans* serovar Lai strain 56601 genome accessible at http://vbc.med.monash.edu.au/genomes [2]. The Paragon method was used in a thorough ID search. The software correction factors provided in the iTRAQ kit were entered in the iTRAQ Isotope Correction Factors table. The detected protein threshold (unused ProtScore) was set at a score of at least 2 (better than 99% confidence). The ProtScore is the sum of the peptide match score calculated as −log_10_[(100-% confidence)/100]. An Unused ProtScore for an identified protein of greater than 2 signifies that there are at least two unique peptides in the data set which match this protein and the confidence for the ID is more than 99%. Detected proteins without quantity ratios were excluded from analysis. Proteins which had an abundance difference of at least 1.5-fold with 95% confidence between the two temperature conditions were considered to be differentially expressed, provided that there was no differential expression between replicates at the same temperature.

## Results and Discussion

Previous studies have found that various leptospiral outer membrane proteins are differentially expressed at different temperatures [8]–[10]. However, our microarray studies did not show any differential expression of some of the corresponding genes at the transcriptional level [7]. Therefore, to complement and enhance the transcriptional study, we evaluated the changes in the protein constituents of the leptospiral outer membrane in response to temperature upshift.

### Proteomic analysis

**Figure 1:**
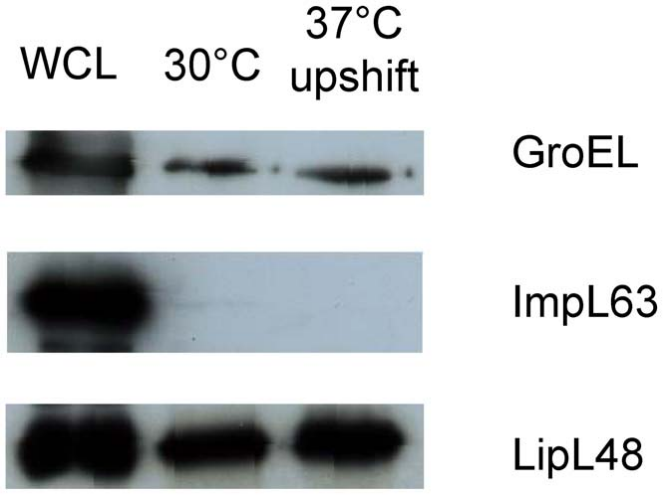
Western immunoblots showing whole cell lysate (WCL) from *L. interrogans* serovar Lai and TX-114 preparations from *L. interrogans* serovar Lai grown at 30°C or upshift to 37°C probed with antisera against GroEL, ImpL63 or LipL48. WCL samples were adjusted for equal protein loading.

Outer membranes (OMs) were extracted using TX-114 from *L. interrogans* grown long-term at 30°C or grown to mid-log phase then shifted to 37°C overnight. Extraction with TX-114 is a validated method of enrichment for leptospiral outer membrane proteins (OMPs) [8],[25],[26],[28]. Immunoblot analysis of the TX-114 preparations failed to detect ImpL63 (inner membrane marker) while the highly abundant cytoplasmic protein, GroEL (cytoplasmic marker), was present in much lower abundance than in whole cell lysate. The OM marker LipL48 was present in high abundance in the TX-114 fraction (Figure 1). This finding indicated that cytoplasmic and inner membrane contamination was minimal in terms of the overall abundance of proteins, although the number subsequently identified by mass spectrometry was large due to high sensitivity of this technique.

**Figure 2:**
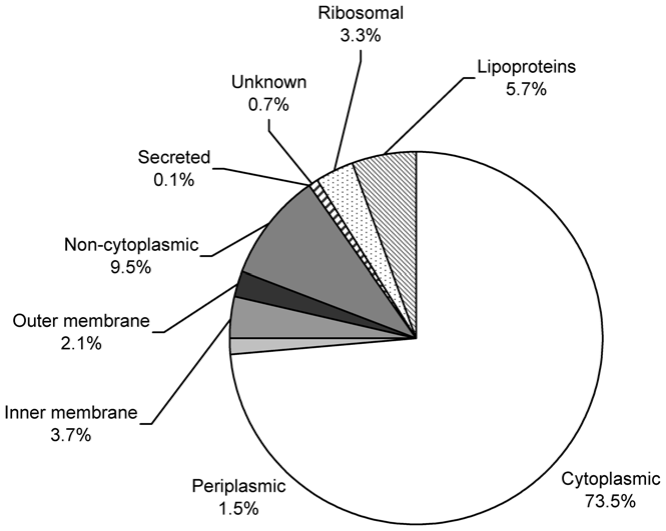
Percentage of leptospiral proteins identified in TX-114 OMP enriched fractions according to location.

Analysis of the output from ProteinPilot revealed a total of 1026 proteins which were identified with 99% confidence (28.4% of proteins predicted in the *L. interrogans* serovar Lai genome). Using the prediction scheme outlined by Bulach *et al.* (2006) [2], 80 of these proteins were predicted or known to be OM located and/or lipoproteins (Table 1). The proportion of proteins identified according to predicted or known location is shown in Figure 2. A total of 256 proteins encoded by the serovar Lai genome has been predicted to be OM located and/or are lipoproteins. Therefore, 26% and 34% of predicted OMPs and lipoproteins respectively were identified in this study. OMPs and lipoproteins comprised only 7.8% of the total proteins identified, a relatively small proportion of the total protein from the sample. Therefore, it appears that while many OMPs were detected, due to the high sensitivity of the 2-DLC/MS-MS analysis method, many other very low abundance proteins from contaminating fractions were also identified, which may not necessarily be detectable by SDS-PAGE, two-dimensional gel electrophoresis (2-DE) or western immunoblot analysis.

**Table 1:** Number of leptospiral proteins identified in the 37°C vs 30°C samples per predicted location. The number of differentially expressed proteins in each location is shown.

Known or predicted location	Number of proteins identified	Down^a^	Up^b^	No change
Cytoplasmic	754	37	19	698
Periplasmic	15	5	0	10
Inner membrane	38	1	1	36
Outer membrane	22	2	1	19
Non-cytoplasmic	97	12	3	82
Secreted	1	0	0	1
Unknown	7	1	0	6
Ribosomal	34	1	0	33
Lipoproteins	58	7	3	48
**Total**	**1026**	**66**	**27**	**933**

^a^≥1.5-fold reduced expression.

^b^≥1.5-fold increased expression.

Recently, it was shown that the TX-114 extraction method is limited in that complete fractionation into the TX-114 detergent phase may not occur for transmembrane proteins and additional localization techniques are required to obtain a comprehensive dataset [29]. Using only the TX-114 extraction method, we identified 31% of predicted OMPs and lipoproteins in this study. A similar study of *Campylobacter jejuni* OMPs using this method identified 87% of predicted membrane proteins [30]. In *L. interrogans* serovar Lai, other predicted OMPs and lipoproteins may not be expressed or present in the membrane under the *in vitro* conditions used. Proteins may be lost during sample processing, not amenable to identification by MS and/or may be expressed at levels below detection limits. For example, the Lk73.5/Sph2 sphingomyelinase (LA1029) protein has been shown to be expressed only under *in vivo* conditions and was not detected in samples from leptospiral cultures grown at 30°C or 37°C [31]. We could likewise not detect this protein. However, it has also been demonstrated that the levels of Sph2 drop considerably at cell densities above 2×10^8^ cells/ml [32] and therefore, the cell density of our samples at time of harvest (3.4×10^8^ and 3.6×10^8^ cells/ml) may have contributed to this effect. LA0695 (LfhA or Lsa24 and recently renamed LenA), a protein which binds factor-H, laminin, collagen IV and fibronectin, was likewise not detected in our study and is known to be expressed during mammalian infections [33]–[35]. Sequence analysis and prediction of tryptic fragments (data not shown) revealed that this 25 kDa protein generates mostly either very large or small peptides, with only 2 predicted in the mass range assayed in this study. LigB, a large OMP (>200 kDa) with immunoglobulin-like domains and associated with virulence [36], was also not detected in our samples. However, LigB is known to be lost upon repeated subculture which is concurrent with loss of virulence [36], although it was recently shown that disruption of *ligB* does not affect virulence of *L. interrogans* in hamsters or rats [37]. The strain of serovar Lai is high passage and no longer virulent in hamsters, which may account for the lack of LigB expression. Alternatively, temperature upshift alone may be insufficient for induction of expression. LigB was likewise not detected in TX-114 preparations in previous studies [8],[9] and is also only partially soluble in TX-100, indicating that it is likely to be distributed between the inner and outer membrane [36]. It is possible that LigB is not soluble in TX-114 and therefore, may be lost during sample processing. Loa22 (LA0222) which has been shown to be a surface-exposed lipoprotein and an essential virulence factor [38],[39] was also not detected in our samples.

Of the identified proteins (Table S1), nine have previously been shown to be surface exposed and located or associated with the outer membrane, namely OmpL1 (LA3138), LipL21 (LA0011), LipL32 (LA2637), LipL36 (LA0492), p31_LipL45_/Qlp42 or LipL45 (LA2295), LipL48 (LA3240), LipL41 (LA0616), LipL46 (LA2024), and LhbpA (LB191) [8],[21],[22],[26],[40],[41],[42],[43],[44],[45]. LipL71/LruA (LA3097) and LruB (LA3469) were also identified in our study. These proteins have been shown to be present mainly in the inner membrane of *L. interrogans* serovar Pomona [46], although LipL71/LruA has been identified in OM preparations from *in vivo* cultivated *L. interrogans* serovar Copenhageni [47]. LruB (pL50) was also identified in OM preparations from *L. interrogans* serovar Lai [8].

Many cytoplasmic enzymes and components of the flagellum were also identified; flagella have previously been detected in outer membrane vesicles of *L. interrogans* [48]. Some proteins may also be transiently located in the outer membrane or complexed with outer membrane proteins and therefore, strict single location may not be the case for a number of proteins. Chaperones in particular are predominantly located in the cytoplasm, but under certain conditions can be membrane-associated in order to assist folding of soluble and membrane proteins. GroEL, GrpE, HtpG, Hsp70 (DnaK) and many other chaperones or heat shock proteins which have been annotated as cytoplasmic proteins were detected in our samples. Notably, GroEL has been shown to be located on the surface of lipid bilayers [49]. Small heat shock proteins together with GroEL may function as a ‘membrane stabilizing factor’ as well as part of a multi-chaperone protein-folding network during thermal stress [50].

### Effects of temperature upshift on protein expression

The relative abundance of each of the proteins between the different temperature conditions (but not different proteins within the same sample) was measured by iTRAQ analysis. Proteins with an expression difference of at least 1.5-fold (50% change in abundance) with 95% confidence between the two temperature conditions were considered to be up- or down-regulated. Of the 1026 proteins identified in our samples, the majority (91%) exhibited no statistically significant change in abundance, while 27 were up-regulated and 66 were down-regulated upon 37°C upshift (Tables 2 and 3 respectively). Consistent with the high proportion of uncharacterized genes in *Leptospira* (50% of coding sequences in the serovar Lai genome), 44% of the differentially expressed proteins currently have no predicted function. This finding is also consistent with comparative genome analyses which suggest that the majority of pathogen-specific genes in *Leptospira* have no ascribed function and the hypothesis that pathogenic *Leptospira* possess unique virulence factors [51],[52]. Since the majority of identified proteins did not exhibit altered abundance at the two temperature conditions, these served as key internal controls for changes in protein expression as well as extraction efficiency between samples. Our data also correlate with expression profiles of previously characterized leptospiral proteins (see below), lending additional confidence to our results and conclusions. Interestingly, recent advances in proteomic analysis have enabled comparisons of the average absolute abundance values of different proteins across samples, and using this approach, it was found that while genes encoding hypothetical proteins comprise more than 40% of the *L. interrogans* genome, the proteins constitute only 12.7% of the total cellular proteins expressed *in vitro* [53].

**Table 2:** Proteins which were at least 1.5-fold up-regulated at 37°C upshift compared to 30°C grouped by COG category. The clusters of orthologous groupings^a^ (COGs) [47] are shown for each protein. The change in transcription at 37°C upshift versus 30°C is shown for comparison with the proteomic data.

Locus tag	Mean fold change	SD	Transcriptional change effect^b^	COG	Predicted location^c^	Gene	Description of gene product
LA1957	2.58	0.28	↑	-	NON-CYT		Hypothetical protein
LA2936	2.41	0.35	↑	-	NON-CYT		LipL45-related lipoprotein
LA2136	2.04	0.19		-	NON-CYT		hypothetical protein
LB242	2.01	0.19		-	NON-CYT		LipL45-related lipoprotein
LA1956	1.95	0.18		-	CYT		Conserved hypothetical protein
LA3807	3.70	0.42		E	CYT	*glnK*	Nitrogen regulatory protein PII
LA2515	2.24	0.26		E	CYT	*hisD*	Histidinol dehydrogenase
LA2790	2.19	0.28		K	CYT		Transcriptional regulator, AcrR-family
LA2483	1.67	0.04		L	CYT	*xerD*	Site-specific recombinase XerD
LA1563	7.32	0.43	↑	O	CYT	*ibpA-2*	Small heat shock protein (molecular chaperone)
LA1564	6.13	0.37		O	CYT	*ibpA-1*	Small heat shock protein (molecular chaperone)
LA1879	2.84	0.09	↑	O	CYT	*clpA-1*	Endopeptidase Clp, ATP-dependent proteolytic subunit
LA3356	2.24	0.09	↑	O	CYT	*gst-1*	glutathione transferase
LA3705	2.22	0.02		O	CYT	*dnaK*	Chaperone protein, Hsp70
LA2312	2.17	0.06		O	NON-CYT		Thiol-disulfide isomerase or thioredoxin
LA2655	2.05	0.28		O	CYT	*groEL*	GroEL chaperone
LA3704	1.93	0.11	↑	O	CYT	*grpE*	Chaperone protein, GrpE
LA2809	1.60	0.03		O	CYT	*ahpC*	Peroxiredoxin
LA1859	3.76	0.06	↑	P	CYT	*katE*	Catalase
LA3598	2.50	0.17	↑	P	CYT	*dps*	DNA-binding ferritin-like protein
LA3242	1.52	0.02		P	OM		TonB-dependent receptor
LA3214	2.18	0.31	↑	S	CYT		Conserved hypothetical protein
LA2295	1.68	0.02		S	IM	*lipL45*	Lipoprotein LipL45
LA3104	2.18	0.15	↑	T	CYT		Signal transduction protein
LB035	2.14	0.13		T	CYT	*wzb*	Protein-tyrosine-phosphatase
LA1526	1.69	0.07		T	CYT		Signal transduction protein containing cAMP-binding and CBS domains
LA3244	1.82	0.02		U	IM	*tolQ*	Transport protein, TolQ-like

^a^Clusters of orthologous groupings are as follows: Information storage and processing (11.2% of coding sequences in *L. interrogans* serovar Lai genome) (includes **J**, translation; **K**, transcription; **L**, replication, recombination and repair); Cellular processes and signalling (19% of coding sequences in the serovar Lai genome) (includes **D**, cell cycle control, cell division, chromosome partitioning; **V**, defence mechanisms; **T**, signal transduction mechanisms; **M**, cell wall, membrane or envelope biogenesis; **N**, cell motility; **U**, intracellular trafficking, secretion and vesicular transport; **O**, post-translational modification, protein turnover, chaperones); Metabolism (18.9% of coding sequences in the serovar Lai genome) (includes **C**, energy production and conversion; **G**, carbohydrate transport and metabolism; **E**, amino acid transport and metabolism; **F**, nucleotide transport and metabolism; **H**, coenzyme transport and metabolism; **I**, lipid transport and metabolism; **P**, inorganic ion transport and metabolism; **Q**, secondary metabolites biosynthesis, transport and catabolism); Poorly characterized (51% of coding sequences in the serovar Lai genome) (includes **R**, general function prediction only; **S**, function unknown; and –, not in COGs).

^b^Transcriptional change at 37°C upshift compared with 30°C [7].

^c^CYT = cytoplasmic; IM = inner membrane; NON-CYT = non-cytoplasmic; OM = outer membrane; PER = periplasmic; UNK = unknown.

**Table 3:** Proteins which were at least 1.5-fold down-regulated at 37°C upshift compared to 30°C grouped by COG category. The COG category is shown for each protein. The change in transcription at 37°C upshift versus 30°C is shown for comparison with the proteomic data.

Locus tag	Mean fold ratio	SD	Mean fold change^a^	Transcriptional change effect^b^	COG^c^	Predicted location^c^	Gene	Description of gene product
LA1402	0.06	0.02	−15.8	↓	-	NON-CYT		Conserved hypothetical protein
LA0715	0.10	0.04	−10.4		-	NON-CYT		hypothetical lipoprotein
LA0858	0.15	0.03	−6.5		-	NON-CYT		Conserved hypothetical lipoprotein
LA1118	0.17	0.02	−5.9		-	NON-CYT		hypothetical protein
LA3814	0.22	0.01	−4.6		-	CYT		Conserved hypothetical protein
LA0419	0.23	0.02	−4.3		-	NON-CYT		hypothetical lipoprotein
LA3769	0.24	0.01	−4.1		-	CYT		hypothetical protein
LA2020	0.25	0.01	−4.0	↓	-	NON-CYT		hypothetical protein
LA4198	0.26	0.01	−3.9	↓	-	CYT		hypothetical protein
LA3379	0.30	0.02	−3.4		-	PER	*flaA-1*	Endoflagellar filament sheath protein
LA2066	0.31	0.04	−3.2		-	NON-CYT		Hypothetical protein
LA3380	0.33	0.01	−3.0		-	PER	*flaA-2*	Endoflagellar filament sheath protein
LA0986	0.34	0.05	−2.9	↑	-	CYT		Conserved hypothetical protein
LA0492	0.35	0.02	−2.9		-	OM	*lipL36*	LipL36, outer membrane lipoprotein
LA1086	0.38	0.01	−2.6		-	NON-CYT		TPR-repeat lipoprotein
LA4208	0.41	0.07	−2.4		-	NON-CYT		Conserved hypothetical protein
LB289	0.42	0.13	−2.4		-	CYT		Hypothetical protein
LA3316	0.42	0.01	−2.4		-	CYT		hypothetical protein
LA4209	0.47	0.05	−2.1		-	NON-CYT		Conserved hypothetical protein
LA3961	0.52	0.05	−1.9		-	NON-CYT		hypothetical protein
LA0117	0.54	0.04	−1.9		-	OM		Conserved hypothetical protein
LA1841	0.55	0.03	−1.8		-	CYT		hypothetical protein
LA1915	0.55	0.06	−1.8		-	OM		TPR-repeat-containing protein
LA1135	0.56	0.06	−1.8		-	CYT		hypothetical protein
LB248	0.57	0.04	−1.8	↓	-	NON-CYT		Conserved hypothetical protein
LA1039	0.58	0.03	−1.7		-	CYT		hypothetical protein
LA0547	0.59	0.03	−1.7		-	CYT		Hypothetical protein
LA2250	0.64	0.01	−1.6	↓	-	CYT		Nuclease S1
LA4224	0.40	0.02	−2.5		C	NON-CYT		FAD-dependent oxidoreductase
LA3470	0.60	0.02	−1.7		C	NON-CYT		Thiol oxidoreductase
LA2197	0.66	0.01	−1.5		C	CYT	*fadH*	2,4-dienoyl-CoA reductase [NADPH]
LA2980	0.36	0.02	−2.7		D	NON-CYT		Conserved hypothetical lipoprotein
LA2570	0.36	0.10	−2.8		E	CYT		Thiamine pyrophosphate-requiring enzyme
LA2145	0.52	0.02	−1.9	↓	E	CYT	*serB*	Phosphoserine phosphatase
LA2360	0.43	0.05	−2.3		F	CYT	*nrdA*	Ribonucleoside-triphosphate reductase, alpha subunit
LA2087	0.54	0.03	−1.8		F	CYT	*guaA-2*	GMP synthase (glutamine-hydrolyzing)
LA0786	0.58	0.04	−1.7	↓	G	CYT		Glycosyltransferase
LA3366	0.23	0.06	−4.4		I	CYT	*caiD-1*	Enoyl-CoA hydratase/carnithine racemase
LA1173	0.57	0.05	−1.8		I	CYT		Acetyl-CoA synthetase
LA0762	0.62	0.03	−1.6		J	CYT	*rpsM*	30S Ribosomal protein S13
LA0258	0.39	0.07	−2.5		L	CYT	*dnaE*	DNA-directed DNA polymerase, alpha subunit
LA2691	0.58	0.03	−1.7	↓	L	CYT		N6-adenine-specific DNA methylase
LA4326	0.48	0.07	−2.1		M	CYT	*lpxD-1*	UDP-3-O-[3-hydroxymyristoyl] glucosamine N-acyltransferase
LA1044	0.55	0.02	−1.8		M	CYT		Conserved hypothetical protein
LA2017	0.39	0.02	−2.6		N	PER	*flaB1*	Endoflagellar filament core protein
LA2418	0.39	0.07	−2.6		N	PER	*flaB-1*	Endoflagellar filament core protein
LA2019	0.57	0.03	−1.8		N	PER	*flaB-2*	Endoflagellar filament core protein
LA1619	0.28	0.00	−3.6		O	CYT		Carbamoyl transferase
LA2194	0.29	0.01	−3.4		O	CYT	*slpA*	Peptidylprolyl isomerase
LA2535	0.44	0.03	−2.3		O	CYT	*ppiB-1*	Peptidylprolyl isomerase
LA3492	0.50	0.05	−2.0		O	NON-CYT		Protease
LA3469	0.37	0.01	−2.7		P	OM	*lruB*	Iron-regulated lipoprotein
LA4016	0.49	0.08	−2.0		Q	CYT		Conserved hypothetical protein
LA3758	0.14	0.02	−7.4	↓	R	CYT		RNA-binding protein
LA2505	0.46	0.05	−2.2		R	CYT		Hydrolase or acyltransferase
LA4300	0.49	0.08	−2.0		R	CYT		ThiJ/PfpI family intracellular protease
LA1671	0.50	0.09	−2.0		R	CYT		Ankyrin repeat protein
LA3028	0.22	0.01	−4.6		S	UNK		Leucine-rich repeat containing protein
LA0985	0.54	0.05	−1.9	↑	S	CYT		Conserved hypothetical protein
LA1983	0.27	0.13	−3.7	↓	T	CYT		Signal transduction protein
LA0599	0.35	0.01	−2.8		T	CYT		Signal transduction protein
LA0049	0.36	0.04	−2.7	↓	T	IM		Methyl-accepting chemotaxis protein
LA0565	0.39	0.04	−2.6	↓	T	CYT		adenylate cyclase
LA2930	0.43	0.03	−2.3		T	CYT		GGDEF domain protein
LB136	0.57	0.03	−1.7	↓	T	CYT		Anti-sigma factor antagonist
LA2267	0.50	0.02	−2.0		U	NON-CYT		conserved hypothetical protein with tetratricopeptide repeat domains

^a^The mean fold change was calculated as the negative inverse of the mean fold ratio.

^b^Transcriptional change at 37°C upshift compared with 30°C [7].

^c^COGs and predicted locations as per Table 2.

### Cell surface and membrane-associated proteins

More OMPs, liproproteins and non-cytoplasmic proteins were down-regulated than up-regulated (Table 1). It is feasible that *L. interrogans* may need to reduce the number of surface proteins at host temperature, perhaps as a strategy for evasion of the host immune response. This possibility is supported by studies showing that expression of various OMPs was reduced in leptospires recovered from guinea pigs [47]. Additionally, in western immunoblot studies of *L. interrogans* excreted in the urine of chronically infected rats compared with *in vitro* grown cultures, serum from infected rats reacted with fewer antigens in *Leptospira* purified from rat urine, suggesting down-regulation of many proteins [54]. In comparative global proteome analyses of *L. interrogans* shifted to 37°C in low-iron medium supplemented with serum, an overall trend was observed towards down-regulation of proteins, especially those involved in energy production, metabolism, regulation and protein synthesis [55]. Up- and down-regulated proteins identified in the present study are listed in Tables 2 and 3 respectively and grouped according to clusters of orthologous groupings (COGs) [56]. Our study correlated with previous findings which showed that LipL36 and LruB are down-regulated, while LipL45 is up-regulated at higher temperatures. LipL36 is an outer membrane lipoprotein which is down-regulated during late-log-phase growth and infection [22],[57] and at temperatures above 30°C [8],[9]. It is also down-regulated under iron-depleted conditions [8]. Therefore, it is likely that LipL36 is involved in survival outside the host. The iron-regulated lipoprotein LruB did not appear to be differentially expressed at 30°C and 37°C in *L. interrogans* serovar Pomona [46] but was found to be down-regulated in *L. interrogans* serovar Lai at 37°C or under iron-limiting conditions in another study (where the protein was identified as pL50) [8]. Our study also found LruB to be down-regulated in *L. interrogans* serovar Lai, thus indicating potential variation in temperature-regulated proteins between different serovars. The peripheral membrane protein LipL45 has been shown to be up-regulated upon upshift to 37°C for 5–7 days [10], while another study found that the protein was up-regulated during late-log-phase growth [21]. There are 11 paralogs of LipL45 encoded by the serovar Lai genome, two of which were also up-regulated with temperature shift (LA2936 and LB242). Another two homologs were not differentially expressed and the remainder were not detected in our samples. It is possible that with 11 paralogs, different proteins are expressed under different environmental conditions or stages of infection, and/or are subject to different regulatory mechanisms. Expression of the surface proteins LipL21, LipL32 and LipL41 was not temperature regulated, consistent with previous studies [8],[9],[26]. Cullen et al [43] also found that the abundance of surface proteins did not alter under different temperature conditions. In contrast, Q8F8Q0 (LA0505) was slightly down-regulated (1.5-fold) upon temperature-upshift in our experiment, possibly due to differences in culture conditions and cell density at harvest. Interestingly, Nally et al [47] found that expression of LipL21 and LipL41 was reduced in *L. interrogans* recovered from infected guinea pigs and therefore, their expression is likely to be regulated by signals other than temperature.

Two predicted peptidylprolyl isomerases (PPIases), LA2194 and LA2535, were down-regulated upon overnight upshift to 37°C. PPIases are thought to contribute to virulence in various bacterial pathogens. For example, the surface-exposed lipoprotein SlrA of *Streptococcus pneumoniae*, is involved in colonization of the nasopharynx, while Mip, a collagen-binding protein in *Legionella pneumophila*, promotes binding and spread of the bacteria through the lungs and spleen [58],[59]. LA2194 and LA2535 may likewise play a similar role in assisting host colonization during leptospiral infection but perhaps are expressed later in the infection process rather than initial establishment of disease, or require additional signals other than temperature for expression.

### Cellular processes and signalling

Several heat stress proteins were up-regulated upon overnight upshift to 37°C, namely small heat shock proteins IbpA-1 and IbpA-2, and the chaperones DnaK (Hsp70), GroEL and GrpE. A previous study found that there was no difference in DnaK or GroEL expression upon upshift to 37°C in *L. interrogans* serovar Pomona, but in that report the cultures were maintained at 37°C for 5–7 days [9]. We found IbpA-2 (LA1563), with a 7.3-fold change, to be the most highly up-regulated protein upon 37°C overnight upshift, while IbpA-1 (LA1564), located immediately downstream of *ibpA-2*, was the second most highly up-regulated protein (6.1-fold). IbpA-2 (or Hsp15) has also been shown in another study to be up-regulated in the early stages of temperature upshift [10] and therefore, may be important in the early stages of infection or adaptation to the host environment. ClpA which performs the ATP-dependent chaperone function of DnaK and DnaJ [60] was also up-regulated 2.8-fold. While these proteins have been predicted to be located in the cytoplasm, membrane association may aid in preserving the structural and functional integrity of the membrane as a short-term mechanism to protect membranes from thermal damage. The chaperones Hsp60 and Hsp70 of *Borrelia burgdorferi* are involved in the molecular processing of flagellin. Their subcellular distribution has been found to be temperature dependent, with relative amounts of membrane-associated protein varying with growth temperatures [61]. DnaK (Hsp70), DnaJ and GrpE have also been shown to be required for the synthesis of flagella in *E. coli* [62] and DnaK is a regulator of flagellar operon expression in *Salmonella enterica* serovar Typhimurium [63].

DnaK is a positive regulator for the expression of all the flagellar regulon genes in *S. enterica*. In *L. interrogans* serovar Lai, the flagellar proteins were down-regulated (the endoflagellar filament core proteins FlaB and filament sheath proteins FlaA). Since some flagellar proteins were down-regulated, but DnaK was up-regulated, the regulatory pathway(s) for leptospiral flagellar synthesis are thus clearly different from those described for *S. enterica*. No other proteins involved in the structure of or synthesis of flagella were differentially expressed. Considerable cellular resources would be needed to synthesize flagella and for motility. Flagellin expression and motility have been found to be switched off in other motile pathogens such as *Yersinia enterocolitica*, *Listeria monocytogenes* and *Bordetella bronchiseptica* upon upshift to 37°C or host entry [64]–[66]. However, while the endoflagellar sheath and filament core proteins were significantly down-regulated in *L. interrogans* serovar Lai upon temperature upshift, we did not observe significant loss of motility. In contrast, Eshghi et al [55] found that proteins involved in motility were up-regulated when leptospires were shifted to low-iron medium supplemented with serum at 37°C. It is likely that *L. interrogans* needs to modulate its motility depending on its environment, during the course of infection and at different host sites.

An enoyl-CoA hydratase (LA3366), involved in fatty acid metabolism, was down-regulated. Lipid metabolism and ultimately lipid composition of the membrane may therefore be altered upon temperature upshift. LpxD-1 (LA4326) was down-regulated 2-fold and interestingly, it has previously been reported that the LPS O antigen (Oag) content of *L. interrogans* recovered from guinea pigs was markedly reduced compared with leptospires cultured *in vitro* [67]. *L. interrogans* possesses a complete set of Lpx proteins which catalyze the biosynthesis of the lipid A anchor of LPS [68],[69]. There was no change in expression of LpxC (LA2306) or LpxD-2 (LA0512), while LpxA (LA3949), LpxB (LA1096) and LpxK (LA3695) were not detected in our samples. However, the genes encoding these proteins are not clustered and therefore not linked transcriptionally. Temperature-regulation of LpxD-1 may therefore be a mechanism for the regulation of LPS synthesis; down-regulation of lipid A synthesis would be a rate-limiting step in Oag synthesis, thus resulting in reduced Oag content during *in vivo* growth.

Stress-related proteins, including Dps (LA3598) (a DNA-binding ferritin-like protein) [70], catalase, glutathione transferase and peroxiredoxin, were up-regulated indicating oxidative stress.

### Transcriptional regulation and signal transduction

Although we did not set out to investigate cytoplasmic regulatory proteins, we found several proteins involved in transcriptional regulation and signal transduction to be differentially regulated upon upshift to 37°C. Pathogens need to be able to detect and respond to different environmental cues. In particular, *L. interrogans* is able to survive under different environmental conditions and encounters many different host environments and defence mechanisms while establishing and maintaining infection. *L. interrogans* has at least 79 two-component regulatory systems, 11 extracytoplasmic function (ECF) sigma factors, 9 anti-sigma factors, 19 anti-sigma factor antagonists, and at least 79 genes involved in motility and chemotaxis [68],[71], thus demonstrating the capability of *L. interrogans* to respond and adapt to a wide range of environmental signals.

Several proteins predicted to be involved in transcriptional regulation, including a protein-tyrosine-phosphatase (LB035), a transcriptional regulator of the AcrR-family (LA2790) and other signal transduction proteins (LA3104 and LA1526) were up-regulated, while other predicted signal transduction proteins (LA0599 and LA1983), a GGDEF domain protein (LA2930), and an anti-sigma factor antagonist (LB136) were down-regulated. Protein-tyrosine-phosphatases are involved in regulating the phosphorylation state of many important signalling molecules, while transcriptional regulators of the AcrR family are transcriptional repressors which respond to stress conditions in *E. coli* [72]. Since several putative transcriptional regulators were identified as being temperature-regulated, these may be involved in the modulation of various signalling pathways leading to expression of virulence or virulence-associated proteins.

### Comparison of proteomic and microarray results

**Figure 3:**
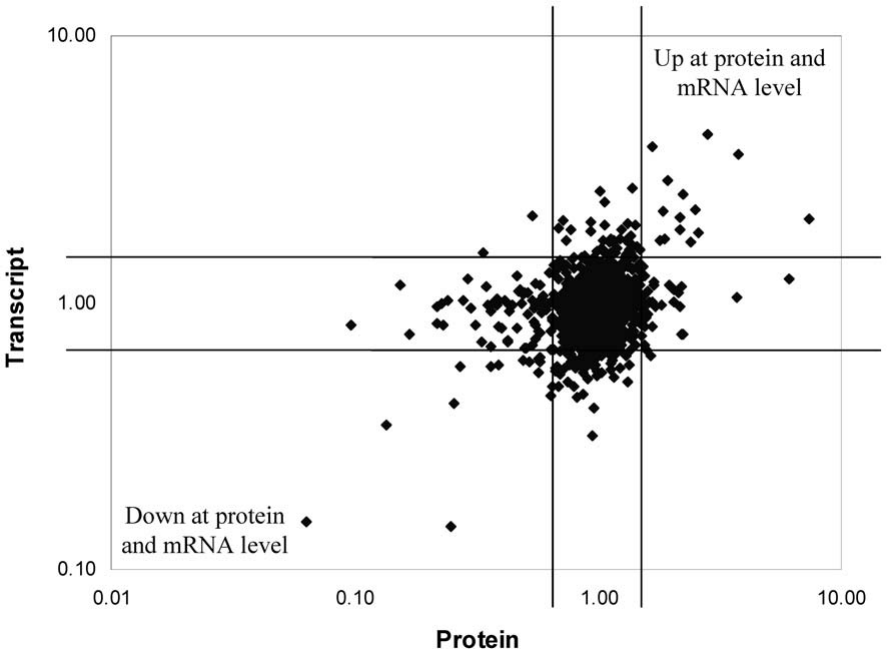
Comparison of protein expression with mRNA expression. Lines indicate 1.5-fold up- or down-regulation at either the mRNA or protein level.

Expression of the 1026 proteins identified was compared to the mRNA expression levels from our previous study [7] (Figure 3). Of the 1026 proteins identified, 93 (9%) were differentially expressed (Tables 2 and 3). Only 25% of the differentially expressed proteins were also differentially expressed (at least 1.5-fold difference) at the mRNA level (*R* ^2^ = 0.12). Conflicting evidence of correlation between mRNA and protein abundance levels has been reported in other studies and in concordance with previous studies of this nature, we also found little correlation between mRNA and protein differential expression [15], [73]–[75]. The heat shock response in the cyanobacterium *Synechocystis* has been investigated by transcriptomic and proteomic approaches and it was found that while there was correlation between the different data sets for major chaperonins and proteases during heat shock, there were areas with no correlation [74]. There remains the very slight possibility that the lack of correlation may partially be the result of having performed protein and transcriptional analyses on different cultures and at different times. However, we believe that is not the reason for our observations, because even in studies where the same cultures have been used for mRNA and protein expression analyses, correlation is still relatively low [73],[74],[76].

The majority of genes/proteins showed no difference in expression at either the transcriptional or translational levels upon overnight temperature upshift (Figure 3). Of the proteins which were significantly differentially expressed (at least 1.5-fold up- or down-regulated with 95% confidence), there were 10 genes/proteins which were up-regulated at both transcriptional and translational levels, 13 which were down-regulated at both transcriptional and translational levels, and 2 where the expression behaviour at mRNA and protein levels were reversed (Figure 3 and Table 4). The small heat shock protein LA1563 was the most highly up-regulated protein upon temperature upshift and was also up-regulated 2-fold at the mRNA level.

**Table 4:** Comparison of proteins and transcripts which were differentially expressed.

Locus tag	COG^a^	Predicted location^a^	Description of gene product
***Protein up, mRNA up***			
LA1563	O	CYT	Small heat shock protein (molecular chaperone)
LA1859	P	CYT	Catalase
LA1879	O	CYT	Endopeptidase Clp, ATP-dependent proteolytic subunit
LA1957	-	NON-CYT	Hypothetical protein
LA2936	-	NON-CYT	LipL45-related lipoprotein
LA3104	T	CYT	Signal transduction protein
LA3214	S	CYT	Conserved hypothetical protein
LA3356	O	CYT	glutathione transferase
LA3598	P	CYT	DNA-binding ferritin-like protein
LA3704	O	CYT	Chaperone protein, GrpE
***Protein down, mRNA down***			
LA0049	T	IM	Methyl-accepting chemotaxis protein
LA0565	T	CYT	adenylate cyclase
LA0786	G	CYT	Glycosyltransferase
LA1402	-	NON-CYT	Conserved hypothetical protein
LA1983	T	CYT	Signal transduction protein
LA2020	-	NON-CYT	hypothetical protein
LA2145	E	CYT	Phosphoserine phosphatase
LA2250	-	CYT	Nuclease S1
LA2691	L	CYT	N6-adenine-specific DNA methylase
LA3758	R	CYT	RNA-binding protein
LA4198	-	CYT	hypothetical protein
LB136	T	CYT	Anti-sigma factor antagonist
LB248	-	NON-CYT	Conserved hypothetical protein
***Protein down, mRNA up***			
LA0985	S	CYT	Conserved hypothetical protein
LA0986	-	CYT	Conserved hypothetical protein

^a^COG categories and predicted locations as in Table 2.

Genes/proteins that were up-regulated at both the mRNA and protein levels reflect the stress that the organism is likely undergoing upon temperature upshift with an overrepresentation of genes encoding heat-stress (*grpE*, *clp* and chaperone genes) and oxidative stress (glutathione transferase and catalase) proteins (Table 4). Other heat shock proteins, DnaK and GroEL were also up-regulated upon temperature upshift but there was no difference in expression at the mRNA level. While transcriptional changes may be rapid and transient, the finding that some proteins are up-regulated with no concurrent change in transcript level suggests longer half-life or greater stability of these transcripts. Interestingly, two genes predicted to encode hypothetical proteins (LA1402 and LA2020) were found to be down-regulated upon temperature upshift at the transcriptional and translational levels, but were found to be two of the most strongly up-regulated transcripts at physiological osmolarity compared with low osmolarity conditions [77]. It was speculated that these genes are most highly expressed during exit from the host in urine into a lower temperature environment [77].

Our temperature microarray studies showed up-regulation of the gene encoding the Lk73.5 sphingomyelinase (LA1029). However, this protein was not detected in our samples in the current study. It is therefore likely that the expression of this and other proteins is regulated at post-transcriptional level. We identified a putative protease, LA3492, which was down-regulated 2-fold upon temperature upshift (Table 3). LA3492 showed similarity to proteases of the prohibitin homology domain family which in mitochondria have been proposed to have a membrane chaperone function [78]. The QmcA protein of *E. coli*, also a prohibitin homology domain family protein, has been proposed to play a role as a membrane chaperone and possibly regulates FtsH, a membrane-bound protease likely to be involved in quality control of membrane proteins [79]. LA3492 may play a similar role in regulation of membrane protein synthesis and folding, and it is feasible that the down-regulation of this putative protease may have been responsible for proteins which showed no change in transcription levels but an increase in protein expression. The lack of correlation between transcript and protein levels of known temperature-regulated proteins LipL36, LruB and LipL45 as well as others, may be due to activities of small non-coding RNAs (sRNAs). Bacteria encode many sRNAs and while most are currently of unknown function, several have been found to modulate post-transcriptional expression of OMPs [11],[12]. There is currently no information on regulatory sRNAs in *Leptospira* spp.

Transcript and protein abundance is likely to be affected by many cellular and physical processes, leading to conflicts in correlation. A study on *Desulfovibrio vulgaris* showed that mRNA abundance alone can explain only 20–28% of the total variation in protein abundance [76]. Other factors include presence of proteases, stability of mRNA, protein translation rates and stability/turnover of proteins. Further studies are needed on transcriptional and translational responses to different environmental signals before we can fully understand the dynamics and interplay of cellular responses.

### Concluding remarks

More OMPs, liproproteins and non-cytoplasmic proteins were down-regulated than up-regulated, suggesting that *L. interrogans* may need to reduce the number of surface proteins at host temperature, perhaps as a host immunity evasion mechanism. The low level of correlation between transcription and translation is intriguing and our data provide a potential basis for further understanding of leptospiral regulatory mechanisms in response to environmental stimuli. Proteomic approaches identify the end stage of gene expression which cannot be determined by mRNA profiling procedures alone. Accordingly, comparative studies on changes in protein expression and changes in transcription on a global basis provide a multidimensional view of the regulation of protein expression in bacteria. Our study provides insight into the changes in protein expression of *Leptospira* during temperature upshift, thus demonstrating the feasibility of this approach, and provides a basis for further comprehensive studies of gene and protein expression changes in response to other conditions such as increase in osmolarity, presence of serum or different iron sources. In this study, the identification of temperature-regulated proteins correlated with studies on previously characterized proteins. However, as with other studies comparing mRNA and protein, we also found low correlation between transcription and translation, indicating that there are many regulatory processes which remain undefined. It is therefore clear that both data sets need to be determined to draw conclusions about changes in protein expression. In the case of *Leptospira*, data are now available on transcriptional changes under different temperature and osmolarity conditions. As data accumulate on transcript and protein expression changes, further comparison and analysis of datasets will yield more knowledge on specific regulatory pathways and cellular events resulting in expression of virulence factors and/or virulence related proteins.

## Supporting Information

Table S1 Supplementary table of all proteins identified.
(1.99 MB DOC)
