# Supplementary material for: Comparative Transcriptional and Translational Analysis of Leptospiral Outer Membrane Protein Expression in Response to Temperature
Source: PLoS Negl Trop Dis. 2009 Dec 8;3(12):e560. doi: 10.1371/journal.pntd.0000560 (PMC2780356; doi:10.1371/journal.pntd.0000560)
Supplement: Table S1 — Supplementary table of all proteins identified. (1.99 MB DOC) [file pntd.0000560.s001.doc]

**Supplementary Table.** Proteins identified in the TX-114 OMP enriched fraction samples.

| **Locus tag** | **Score*^a^*** | **% Coverage*^b^*** | **Mean fold change*^c^*** | **SD** | **COG*^d^*** | **Predicted location*^d^*** | **Gene** | **Description of gene product** |
| --- | --- | --- | --- | --- | --- | --- | --- | --- |
| LA_SPN1928 | 8.00 | 59.86 | 0.74 | 0.11 | - | NON-CYT |  | Conserved hypothetical lipoprotein |
| LA0002 | 30.38 | 53.08 | 1.15 | 0.02 | L | CYT | *dnaN* | DNA-directed DNA polymerase, beta subunit |
| LA0005 | 13.40 | 21.91 | 0.94 | 0.07 | L | CYT | *gyrB* | DNA gyrase subunit B |
| LA0009 | 11.17 | 38.78 | 1.30 | 0.05 | - | NON-CYT |  | Hypothetical lipoprotein |
| LA0011 | 2.00 | 11.83 | 1.03 | 0.20 | - | OM | *lipL21* | LipL21 lipoprotein |
| LA0018 | 4.00 | 24.16 | 1.29 | 0.06 | - | NON-CYT |  | hypothetical protein |
| LA0020 | 11.76 | 32.79 | 1.19 | 0.08 | R | CYT |  | Short chain dehydrogenase |
| LA0025 | 2.00 | 16.02 | 0.53 | 0.11 | N | CYT | *fliG-1* | Endoflagellar motor switch protein |
| LA0031 | 2.49 | 18.80 | 0.98 | 0.07 | S | IM |  | Conserved hypothetical protein |
| LA0032 | 21.00 | 53.19 | 1.10 | 0.03 | - | NON-CYT |  | hypothetical lipoprotein |
| LA0033 | 5.30 | 39.50 | 1.25 | 0.09 | G | CYT |  | Inositol monophosphatase family protein |
| LA0034 | 2.92 | 9.57 | 1.55 | 0.42 | - | CYT |  | hypothetical protein |
| LA0036 | 4.00 | 21.05 | 0.74 | 0.11 | O | CYT |  | HSP33-family chaperone |
| LA0039 | 4.96 | 18.14 | 1.42 | 0.28 | O | CYT |  | Metal-dependent molecular chaperone |
| LA0043 | 5.62 | 16.33 | 1.49 | 0.07 | R | NON-CYT |  | TPR-repeat-containing protein |
| LA0045 | 2.00 | 11.97 | 1.14 | 0.16 | E | CYT | *aroD* | 3-dehydroquinate dehydratase |
| LA0046 | 14.03 | 40.68 | 1.14 | 0.04 | M | CYT |  | Sugar phosphate isomerase |
| LA0049 | 4.49 | 12.74 | 0.36 | 0.04 | T | IM |  | Methyl-accepting chemotaxis protein |
| LA0051 | 18.67 | 51.28 | 0.87 | 0.02 | M | CYT |  | Nucleoside-diphosphate-sugar epimerase |
| LA0052 | 43.31 | 61.18 | 1.17 | 0.02 | C | IM | *pntA* | NAD(P)(+) transhydrogenase (AB-specific), alpha subunit |
| LA0054 | 24.48 | 44.42 | 1.02 | 0.03 | J | CYT | *hisS* | Histidine--tRNA ligase |
| LA0067 | 5.03 | 13.48 | 0.84 | 0.15 | T | IM |  | Serine phosphatase RsbU, regulator of sigma subunit |
| LA0072 | 6.12 | 39.88 | 0.96 | 0.05 | F | CYT | *dcd* | dCTP deaminase |
| LA0076 | 8.20 | 82.79 | 1.01 | 0.03 | E | CYT |  | Dioxygenase superfamily protein |
| LA0082 | 3.70 | 20.49 | 1.33 | 0.09 | H | CYT |  | Methylase/methyltransferase |
| LA0083 | 8.18 | 25.27 | 1.31 | 0.03 | E | CYT | *dapF* | Diaminopimelate epimerase |
| LA0097 | 2.00 | 6.76 | 1.49 | 0.21 | - | IM |  | hypothetical protein |
| LA0104 | 2.00 | 11.23 | 0.94 | 0.22 | R | NON-CYT |  | Zn-dependent hydrolase |
| LA0106 | 6.00 | 14.64 | 1.53 | 0.18 | I | CYT | *fadD* | Long-chain-fatty-acid--CoA ligase |
| LA0110 | 2.00 | 7.61 | 1.40 | 0.37 | E | CYT |  | Para-aminobenzoate synthase |
| LA0113 | 6.63 | 11.60 | 0.91 | 0.06 | E | CYT |  | Cys/Met metabolism PLP-dependent protein |
| LA0114 | 9.50 | 28.25 | 0.92 | 0.02 | J | CYT | *prfA* | Peptide chain release factor 1 |
| LA0117 | 2.46 | 24.12 | 0.54 | 0.04 | - | OM |  | Conserved hypothetical protein |
| LA0119 | 3.79 | 20.14 | 1.15 | 0.04 | - | CYT |  | Hypothetical protein |
| LA0126 | 10.79 | 46.89 | 1.46 | 0.08 | E | CYT | *hisA* | 1-(5-phosphoribosyl)-5-[(5-phosphoribosylamino)methylideneamino] imidazole-4-carboxamide isomerase |
| LA0127 | 10.00 | 21.78 | 1.24 | 0.03 | O | IM |  | Protein-disulfide isomerase |
| LA0129 | 6.00 | 18.95 | 0.97 | 0.11 | S | NON-CYT |  | Conserved hypothetical protein |
| LA0130 | 6.00 | 18.68 | 0.90 | 0.16 | H | CYT | *thiD* | Phosphomethylpyrimidine kinase |
| LA0136 | 15.63 | 52.10 | 1.16 | 0.05 | S | NON-CYT |  | LipL45-related lipoprotein |
| LA0137 | 29.03 | 48.98 | 0.79 | 0.03 | - | NON-CYT |  | Hypothetical lipoprotein |
| LA0138 | 40.53 | 32.83 | 0.76 | 0.01 | - | NON-CYT |  | TPR-repeat-containing lipoprotein |
| LA0141 | 10.38 | 25.96 | 1.18 | 0.03 | R | CYT |  | Metal-dependent hydrolase of the beta-lactamase superfamily I |
| LA0146 | 21.00 | 36.48 | 0.96 | 0.08 | K | CYT |  | Transcriptional regulator |
| LA0147 | 6.00 | 7.35 | 0.57 | 0.06 | - | CYT |  | Conserved hypothetical protein |
| LA0151 | 2.00 | 16.67 | 1.24 | 0.04 | O | CYT |  | Conserved hypothetical protein |
| LA0153 | 20.89 | 42.13 | 1.04 | 0.06 | T | CYT |  | Conserved hypothetical protein |
| LA0157 | 6.78 | 27.11 | 1.05 | 0.03 | E | CYT | *aroC* | Chorismate synthase |
| LA0160 | 10.82 | 25.73 | 0.81 | 0.06 | C | CYT | *nuoG* | NADH dehydrogenase (ubiquinone), G chain |
| LA0169 | 8.04 | 38.53 | 0.74 | 0.04 | Q | CYT |  | Dithiol-disulfide isomerase involved in polyketide biosynthesis |
| LA0171 | 8.01 | 28.10 | 1.13 | 0.11 | E | CYT | *avtA* | Valine--pyruvate transaminase |
| LA0178 | 17.38 | 23.92 | 1.00 | 0.03 | U | IM | *yidC* | Preprotein translocase, YidC subunit |
| LA0179 | 12.94 | 47.88 | 0.71 | 0.01 | R | CYT |  | Jag-like RNA-binding protein |
| LA0180 | 5.52 | 24.78 | 0.84 | 0.02 | R | CYT | *trmE* | tRNA modification GTPase TrmE |
| LA0181 | 13.36 | 28.38 | 0.98 | 0.04 | C | CYT | *sfcA* | Malate dehydrogenase (oxaloacetate decarboxylating) |
| LA0184 | 11.72 | 30.49 | 1.02 | 0.04 | - | CYT |  | hypothetical protein |
| LA0185 | 17.58 | 39.87 | 1.05 | 0.02 | C | CYT | *fumC* | Fumarate hydratase |
| LA0195 | 2.47 | 38.64 | 1.45 | 0.34 | - | CYT |  | Conserved hypothetical protein |
| LA0224 | 4.00 | 16.79 | 1.53 | 0.08 | R | CYT |  | Amidohydrolase |
| LA0243 | 4.47 | 6.74 | 1.33 | 0.12 | C | IM | *cyoB* | Cytochrome C oxidase, subunit I |
| LA0249 | 4.00 | 29.28 | 0.74 | 0.23 | K | CYT |  | Transcriptional regulator |
| LA0251 | 47.23 | 55.85 | 0.95 | 0.01 | C | CYT | *pckA* | Phosphoenolpyruvate carboxykinase (ATP) |
| LA0253 | 2.00 | 6.50 | 1.40 | 0.29 | S | NON-CYT |  | Conserved hypothetical lipoprotein |
| LA0255 | 6.00 | 14.73 | 0.80 | 0.05 | D | CYT |  | ParA-like protein |
| LA0256 | 30.78 | 47.12 | 1.10 | 0.04 | J | CYT | *gatB* | Aspartyl/glutamyl-tRNA(Asn/Gln) amidotransferase subunit B |
| LA0258 | 4.01 | 6.14 | 0.39 | 0.07 | L | CYT | *dnaE* | DNA-directed DNA polymerase, alpha subunit |
| LA0261 | 5.17 | 18.94 | 1.01 | 0.04 | K | CYT |  | RNA polymerase sigma subunit |
| LA0263 | 19.94 | 56.61 | 0.94 | 0.09 | - | CYT |  | Hypothetical protein |
| LA0274 | 2.00 | 18.22 | 1.04 | 0.18 | V | IM | *lolD-2* | Lipoprotein releasing system, LolD ATPase component |
| LA0280 | 28.14 | 52.59 | 1.04 | 0.03 | T | CYT |  | cAMP-binding protein |
| LA0284 | 6.63 | 25.11 | 0.89 | 0.12 | - | CYT |  | hypothetical protein |
| LA0292 | 2.00 | 11.96 | 0.96 | 0.17 | J | CYT | *trmA-2* | tRNA (uracil-5-)-methyltransferase |
| LA0293 | 2.00 | 14.40 | 0.83 | 0.17 | L | CYT | *sbcC* | ATPase involved in DNA repair |
| LA0296 | 19.39 | 47.47 | 0.97 | 0.05 | R | CYT |  | Zinc-binding dehydrogenase |
| LA0298 | 4.00 | 10.65 | 1.06 | 0.12 | - | CYT |  | hypothetical protein |
| LA0301 | 5.67 | 14.94 | 0.83 | 0.18 | M | OM |  | OmpA-family protein |
| LA0302 | 2.00 | 26.75 | 1.07 | 0.21 | - | CYT |  | Conserved hypothetical protein |
| LA0309 | 5.52 | 24.89 | 1.04 | 0.05 | T | CYT |  | cAMP-binding protein, regulatory protein |
| LA0310 | 12.02 | 31.34 | 1.10 | 0.03 | T | CYT |  | cAMP-binding protein, regulatory protein |
| LA0311 | 2.00 | 7.69 | 1.01 | 0.22 | F | CYT |  | Metal-dependent hydrolase |
| LA0313 | 53.97 | 55.95 | 0.94 | 0.02 | J | CYT |  | Protein-synthesizing GTPase complex, EF-G component |
| LA0325 | 6.09 | 21.18 | 1.24 | 0.06 | C | CYT |  | Zn-dependent oxidoreductase |
| LA0327 | 2.00 | 15.83 | 1.30 | 0.22 | J | CYT |  | rRNA methylase/Hemolysin-tylA |
| LA0335 | 12.01 | 26.82 | 1.14 | 0.12 | C | IM | *pntB* | NAD(P)(+) transhydrogenase , beta component |
| LA0339 | 9.43 | 34.06 | 1.24 | 0.08 | C | NON-CYT | *glpQ* | Glycerophosphodiester phosphodiesterase |
| LA0343 | 2.00 | 15.56 | 1.04 | 0.09 | S | CYT |  | Conserved hypothetical protein |
| LA0351 | 2.00 | 20.06 | 1.16 | 0.24 | U | NON-CYT |  | TPR-repeat-containing protein |
| LA0360 | 18.63 | 19.50 | 0.91 | 0.05 | E | CYT | *gcvP* | Glycine dehydrogenase (decarboxylating), protein P |
| LA0362 | 18.66 | 51.48 | 1.00 | 0.04 | E | CYT | *gcvT* | Aminomethyltransferase |
| LA0365 | 11.59 | 23.69 | 1.06 | 0.03 | - | NON-CYT |  | conserved hypothetical protein |
| LA0366 | 15.10 | 41.07 | 1.21 | 0.08 | E | CYT | *serC* | Phosphoserine transaminase |
| LA0370 | 8.66 | 18.05 | 1.44 | 0.06 | - | NON-CYT |  | Conserved hypothetical protein |
| LA0376 | 2.00 | 7.12 | 1.03 | 0.15 | - | CYT |  | Conserved hypothetical protein |
| LA0391 | 34.35 | 42.43 | 0.72 | 0.02 | O | CYT | *clpA-3* | ATP-dependent Clp protease |
| LA0400 | 5.70 | 30.59 | 0.74 | 0.05 | - | CYT |  | Hypothetical protein |
| LA0401 | 8.00 | 55.00 | 1.02 | 0.03 | T | CYT | *sixA-1* | Phosphohistidine phosphatase |
| LA0402 | 4.07 | 30.92 | 0.99 | 0.25 | V | CYT |  | ATP-binding protein of an ABC transporter complex |
| LA0404 | 7.00 | 18.49 | 0.72 | 0.11 | T | CYT |  | Signal transduction protein |
| LA0408 | 5.10 | 14.91 | 1.46 | 0.31 | J | CYT | *trpS* | Tryptophan--tRNA ligase |
| LA0409 | 16.42 | 35.48 | 1.23 | 0.03 | - | CYT |  | hypothetical protein |
| LA0411 | 62.15 | 95.92 | 1.12 | 0.02 | C | CYT | *etfA* | Electron transfer flavoprotein, alpha subunit |
| LA0412 | 35.50 | 84.98 | 1.16 | 0.03 | C | CYT | *eftB* | Electron transfer flavoprotein, beta subunit |
| LA0414 | 16.89 | 42.89 | 0.83 | 0.07 | I | CYT | *ivd* | Isovaleryl-CoA dehydrogenase |
| LA0419 | 2.00 | 10.99 | 0.23 | 0.02 | - | NON-CYT |  | hypothetical lipoprotein |
| LA0421 | 2.00 | 8.33 | 0.74 | 0.11 | - | UNK |  | hypothetical protein |
| LA0433 | 7.58 | 30.94 | 1.35 | 0.09 | L | NON-CYT |  | Conserved hypothetical protein |
| LA0439 | 9.52 | 19.34 | 0.87 | 0.03 | G | CYT | *gpmI* | Phosphoglycerate mutase |
| LA0441 | 2.01 | 15.77 | 0.59 | 0.19 | - | CYT |  | Hypothetical protein |
| LA0448 | 6.06 | 18.58 | 0.94 | 0.14 | R | IM |  | ATP-binding protein of an ABC transporter complex |
| LA0449 | 6.00 | 37.84 | 1.21 | 0.04 | P | CYT |  | Rhodanese-related sulfurtransferase |
| LA0457 | 33.78 | 66.67 | 1.07 | 0.05 | I | CYT | *paaJ-4* | Acetyl-CoA C-acetyltransferase |
| LA0459 | 4.03 | 10.79 | 0.86 | 0.07 | - | NON-CYT |  | Hypothetical lipoprotein |
| LA0462 | 8.00 | 43.77 | 0.81 | 0.11 | - | CYT |  | hypothetical protein |
| LA0464 | 4.00 | 34.78 | 1.13 | 0.15 | K | CYT | *nusB* | Transcription antitermination protein |
| LA0466 | 11.24 | 23.77 | 0.79 | 0.04 | C | NON-CYT |  | Conserved hypothetical protein |
| LA0484 | 22.55 | 45.11 | 1.05 | 0.08 | K | CYT |  | Transcriptional regulator |
| LA0492 | 10.00 | 29.23 | 0.35 | 0.02 | - | OM | *lipL36* | LipL36, outer membrane lipoprotein |
| LA0494 | 7.10 | 24.68 | 0.81 | 0.05 | - | NON-CYT |  | hypothetical lipoprotein |
| LA0505 | 20.67 | 48.91 | 0.65 | 0.02 | - | NON-CYT |  | Conserved hypothetical protein |
| LA0506 | 2.01 | 28.89 | 1.16 | 0.14 | - | NON-CYT |  | Hypothetical protein |
| LA0512 | 6.00 | 17.94 | 1.48 | 0.19 | M | CYT | *lpxD-2* | UDP-3-O-[3-hydroxymyristoyl] glucosamine N-acyltransferase |
| LA0520 | 6.00 | 23.81 | 0.82 | 0.07 | S | IM |  | Conserved hypothetical protein |
| LA0532 | 8.02 | 26.60 | 0.68 | 0.04 | - | NON-CYT |  | hypothetical protein |
| LA0533 | 8.00 | 24.80 | 1.12 | 0.06 | M | CYT |  | Pyridoxal phosphate-dependent aminotransferase |
| LA0538 | 4.00 | 12.39 | 1.06 | 0.14 | K | CYT |  | Transcriptional regulator, ThiJ/PfpI family protein |
| LA0546 | 28.78 | 36.15 | 1.00 | 0.06 | T | CYT |  | cAMP-dependent protein kinase |
| LA0547 | 4.00 | 20.61 | 0.59 | 0.03 | - | CYT |  | Hypothetical protein |
| LA0549 | 2.01 | 10.29 | 0.81 | 0.07 | - | CYT |  | Hypothetical protein |
| LA0551 | 3.70 | 20.86 | 1.17 | 0.14 | E | CYT | *hisI* | Phosphoribosyl-AMP cyclohydrolase |
| LA0560 | 44.20 | 66.97 | 0.93 | 0.03 | I | CYT |  | Acyl-CoA dehydrogenase |
| LA0565 | 7.69 | 19.08 | 0.39 | 0.04 | T | CYT |  | adenylate cyclase |
| LA0568 | 2.00 | 6.98 | 0.95 | 0.15 | I | OM |  | Outer membrane transport protein |
| LA0595 | 4.00 | 16.35 | 1.26 | 0.05 | O | CYT | *gst-3* | Glutathione S-transferase |
| LA0599 | 6.92 | 30.94 | 0.35 | 0.01 | T | CYT |  | Signal transduction protein |
| LA0611 | 15.70 | 23.72 | 1.03 | 0.04 | D | CYT | *ftsA* | Cell division protein, actin-like ATPase |
| LA0612 | 10.00 | 21.00 | 0.89 | 0.18 | D | CYT | *ftsZ* | Cell division GTPase |
| LA0616 | 50.24 | 73.52 | 0.92 | 0.02 | - | OM | *lipL41* | LipL41 lipoprotein |
| LA0618 | 6.80 | 22.84 | 1.34 | 0.22 | H | CYT | *nadA* | Quinolinate synthase, Protein A |
| LA0630 | 8.00 | 21.24 | 0.98 | 0.11 | C | CYT |  | Oxidoreductase |
| LA0644 | 2.00 | 24.02 | 1.14 | 0.04 | - | NON-CYT |  | hypothetical protein |
| LA0645 | 7.05 | 20.21 | 1.13 | 0.08 | - | CYT |  | Conserved hypothetical protein |
| LA0646 | 14.93 | 35.68 | 1.07 | 0.06 | J | CYT | *rpsA-1* | 30S Ribosomal protein S1 |
| LA0648 | 7.13 | 25.23 | 1.25 | 0.14 | T | CYT |  | Anti-sigma regulatory factor |
| LA0649 | 18.12 | 33.33 | 1.14 | 0.08 | L | CYT | *uvrB* | Helicase subunit of the DNA excision repair complex |
| LA0653 | 11.40 | 71.82 | 1.32 | 0.09 | T | CYT |  | Anti-sigma factor antagonist |
| LA0656 | 4.60 | 19.09 | 1.52 | 0.13 | G | CYT | *manA* | Mannose-6-phosphate isomerase |
| LA0665 | 2.00 | 23.05 | 0.89 | 0.30 | R | CYT |  | Zinc-dependant hydrolase |
| LA0671 | 4.00 | 16.67 | 1.14 | 0.08 | C | CYT | *gltA-2* | Citrate synthase |
| LA0674 | 12.16 | 23.54 | 1.03 | 0.08 | R | CYT |  | Oxidoreductase |
| LA0675 | 12.40 | 32.60 | 1.13 | 0.01 | - | NON-CYT |  | Conserved hypothetical protein |
| LA0678 | 6.80 | 15.61 | 1.26 | 0.15 | T | NON-CYT |  | Methyl-accepting chemotaxis protein |
| LA0693 | 19.41 | 52.84 | 0.80 | 0.04 | E | CYT | *lysC* | Aspartate kinase |
| LA0710 | 4.57 | 29.73 | 1.20 | 0.23 | - | NON-CYT |  | hypothetical lipoprotein |
| LA0715 | 2.00 | 7.98 | 0.10 | 0.04 | - | NON-CYT |  | hypothetical lipoprotein |
| LA0720 | 10.28 | 39.36 | 1.31 | 0.06 | S | CYT |  | Conserved hypothetical protein |
| LA0727 | 32.05 | 32.64 | 1.04 | 0.08 | E | CYT | *carB* | Carbamoyl-phosphate synthase (glutamine-hydrolyzing), large subunit |
| LA0736 | 6.05 | 18.20 | 1.01 | 0.04 | J | CYT |  | Protein-synthesizing GTPase complex, EF-G component |
| LA0737 | 70.30 | 84.04 | 0.94 | 0.01 | J | CYT | *tufB* | Protein-synthesizing GTPase complex, EF-Tu component |
| LA0740 | 11.54 | 36.97 | 0.76 | 0.03 | J | CYT | *rplD* | 50S Ribosomal protein L4 |
| LA0741 | 8.39 | 52.88 | 0.91 | 0.03 | J | CYT | *rplW* | 50S Ribosomal protein L23 |
| LA0742 | 16.15 | 55.91 | 0.70 | 0.01 | J | CYT | *rplB* | 50S Ribosomal protein L2 |
| LA0744 | 7.70 | 55.45 | 0.81 | 0.07 | J | CYT | *rplV* | 50S Ribosomal protein L22 |
| LA0745 | 7.76 | 48.00 | 0.70 | 0.07 | J | CYT | *rpsC* | 30S Ribosomal protein S3 |
| LA0746 | 5.05 | 46.72 | 0.92 | 0.05 | J | CYT | *rplP* | 50S Ribosomal protein L16 |
| LA0747 | 4.00 | 18.09 | 1.00 | 0.05 | J | CYT | *rpmC* | 50S Ribosomal protein L29 |
| LA0748 | 5.52 | 31.46 | 0.62 | 0.04 | J | CYT | *rpsQ* | 30S Ribosomal protein S17 |
| LA0751 | 25.62 | 71.43 | 0.89 | 0.04 | J | CYT | *rplE* | 50S Ribosomal protein L5 |
| LA0753 | 6.52 | 33.08 | 0.72 | 0.03 | J | CYT | *rpsH* | 30S Ribosomal protein S8 |
| LA0754 | 19.08 | 71.51 | 0.96 | 0.03 | J | CYT | *rplF* | 50S Ribosomal protein L6 |
| LA0755 | 5.08 | 22.95 | 0.79 | 0.03 | J | CYT | *rplR* | 50S Ribosomal protein L18 |
| LA0756 | 8.02 | 33.33 | 0.80 | 0.04 | J | CYT | *rpsE* | 30S Ribosomal protein S5 |
| LA0758 | 5.03 | 23.89 | 0.91 | 0.03 | J | CYT | *rplO* | 50S Ribosomal protein L15 |
| LA0760 | 6.00 | 22.99 | 1.12 | 0.03 | F | CYT | *adk* | Adenylate kinase |
| LA0762 | 4.54 | 46.40 | 0.62 | 0.03 | J | CYT | *rpsM* | 30S Ribosomal protein S13 |
| LA0764 | 11.43 | 47.34 | 0.62 | 0.04 | J | CYT | *rpsD* | 30S Ribosomal protein S4 |
| LA0766 | 5.22 | 28.65 | 0.89 | 0.04 | J | CYT | *rplQ* | 50S Ribosomal protein L17 |
| LA0776 | 39.27 | 70.83 | 0.96 | 0.02 | E | CYT |  | Aspartate/tyrosine/aromatic aminotransferase |
| LA0778 | 11.38 | 33.44 | 0.90 | 0.04 | F | CYT | *pyrB* | Aspartate carbamoyltransferase, catalytic chain |
| LA0785 | 10.40 | 26.22 | 1.40 | 0.18 | R | CYT |  | Zn-dependent hydrolase |
| LA0786 | 4.00 | 15.51 | 0.58 | 0.04 | G | CYT |  | Glycosyltransferase |
| LA0787 | 3.70 | 8.39 | 0.75 | 0.10 | J | CYT |  | rRNA methylase |
| LA0790 | 10.13 | 24.24 | 1.16 | 0.06 | C | CYT | *gltA-1* | Citrate (Si)-synthase |
| LA0791 | 10.27 | 23.90 | 0.74 | 0.05 | M | PER |  | Transglycosylase |
| LA0799 | 6.44 | 41.10 | 0.74 | 0.06 | M | UNK |  | Shape determination protein |
| LA0800 | 10.02 | 34.09 | 1.09 | 0.05 | H | CYT | *pdxA* | 4-hydroxythreonine-4-phosphate dehydrogenase |
| LA0809 | 16.09 | 59.79 | 1.38 | 0.08 | O | PER |  | Trypsin-like serine protease |
| LA0810 | 8.01 | 27.57 | 0.85 | 0.03 | L | CYT | *ruvB* | Holliday junction DNA helicase |
| LA0825 | 4.00 | 20.10 | 1.20 | 0.05 | K | CYT |  | Transcriptional regulator, AcrR-family |
| LA0827 | 12.81 | 41.61 | 0.88 | 0.03 | R | CYT |  | thioesterase |
| LA0828 | 58.65 | 85.03 | 0.77 | 0.03 | I | CYT | *paaJ-1* | Acetyl-CoA acetyltransferase |
| LA0832 | 4.00 | 13.10 | 0.94 | 0.04 | H | CYT | *birA* | Biotin--[acetyl-CoA-carboxylase] ligase |
| LA0833 | 4.23 | 24.12 | 0.93 | 0.04 | K | CYT |  | Transcriptional regulator |
| LA0838 | 6.30 | 15.77 | 0.95 | 0.04 | M | CYT | *murD* | UDP-N-acetylmuramoylalanine--D-glutamate ligase |
| LA0839 | 13.05 | 61.82 | 1.38 | 0.07 | T | CYT |  | Antisigma factor antagonist |
| LA0840 | 7.40 | 18.25 | 1.26 | 0.15 | E | CYT | *trpC* | Indole-3-glycerol-phosphate synthase |
| LA0845 | 4.00 | 20.13 | 1.17 | 0.13 | E | CYT | *leuA-4* | 2-isopropylmalate synthase |
| LA0851 | 4.39 | 52.38 | 0.83 | 0.08 | J | CYT | *rpmA* | 50S Ribosomal protein L27 |
| LA0854 | 30.23 | 45.67 | 1.28 | 0.01 | E | CYT | *proA* | Glutamate-5-semialdehyde dehydrogenase |
| LA0855 | 2.00 | 17.59 | 0.56 | 0.19 | H | CYT | *nadD* | Nicotinic acid mononucleotide adenylyltransferase |
| LA0858 | 2.00 | 3.25 | 0.15 | 0.03 | - | NON-CYT |  | Conserved hypothetical lipoprotein |
| LA0861 | 4.00 | 34.33 | 1.05 | 0.05 | T | CYT |  | Anti-sigma factor antagonist |
| LA0862 | 11.72 | 54.39 | 0.78 | 0.02 | O | CYT | *tpx* | Peroxiredoxin |
| LA0869 | 2.35 | 23.41 | 0.80 | 0.07 | C | CYT |  | NAD-dependent aldehyde dehydrogenase |
| LA0891 | 4.00 | 26.42 | 1.18 | 0.24 | C | CYT | *nuoE* | NADH dehydrogenase (ubiquinone), E chain |
| LA0892 | 6.77 | 16.79 | 1.02 | 0.06 | C | CYT | *nuoD* | NADH dehydrogenase (ubiquinone), D chain |
| LA0899 | 12.77 | 56.49 | 0.87 | 0.04 | I | CYT |  | Acyl dehydratase |
| LA0903 | 7.47 | 14.55 | 1.12 | 0.13 | V | CYT |  | Methylase |
| LA0912 | 10.01 | 24.81 | 0.87 | 0.08 | R | NON-CYT |  | TPR-repeat protein |
| LA0913 | 24.75 | 46.89 | 1.23 | 0.07 | S | OM |  | NHL repeat protein |
| LA0919 | 4.00 | 26.86 | 0.85 | 0.13 | - | CYT |  | hypothetical protein |
| LA0929 | 4.13 | 29.91 | 1.01 | 0.10 | C | CYT |  | Enolase-phosphatase |
| LA0942 | 47.64 | 82.61 | 0.99 | 0.03 | K | CYT | *nusA* | Transcription elongation factor |
| LA0943 | 22.89 | 36.04 | 0.70 | 0.03 | J | CYT | *infB* | Translation initiation factor 2 (IF-2; GTPase) |
| LA0945 | 2.04 | 23.67 | 1.04 | 0.20 | J | CYT | *truB* | Pseudouridylate synthase, N-terminal subunit |
| LA0946 | 3.30 | 45.45 | 0.72 | 0.01 | J | CYT | *rpsO* | 30S Ribosomal protein S15 |
| LA0947 | 13.21 | 29.23 | 0.96 | 0.04 | J | CYT | *pnp* | Polyribonucleotide nucleotidyltransferase |
| LA0954 | 17.34 | 30.61 | 1.25 | 0.09 | - | CYT |  | hypothetical protein |
| LA0956 | 73.85 | 45.99 | 1.00 | 0.02 | E | CYT | *gltB* | Glutamate synthase (NADH) |
| LA0957 | 18.85 | 42.91 | 0.98 | 0.03 | U | OM |  | Outer membrane efflux protein related to TolC |
| LA0960 | 7.70 | 43.33 | 1.36 | 0.22 | - | NON-CYT |  | Conserved hypothetical protein |
| LA0969 | 4.11 | 23.79 | 0.76 | 0.07 | V | CYT |  | ATP-binding protein of an ABC transporter complex |
| LA0985 | 4.87 | 18.57 | 0.54 | 0.05 | S | CYT |  | Conserved hypothetical protein |
| LA0986 | 2.00 | 5.25 | 0.34 | 0.05 | - | CYT |  | Conserved hypothetical protein |
| LA1008 | 3.00 | 16.78 | 1.08 | 0.11 | S | CYT |  | Conserved hypothetical protein |
| LA1010 | 2.27 | 11.18 | 1.12 | 0.03 | - | NON-CYT |  | hypothetical protein |
| LA1019 | 9.10 | 31.18 | 0.87 | 0.09 | S | CYT |  | Conserved hypothetical protein |
| LA1021 | 14.44 | 29.67 | 0.85 | 0.04 | K | CYT | *rho* | Transcription termination factor Rho |
| LA1036 | 14.84 | 32.03 | 1.05 | 0.05 | T | CYT |  | Histidine kinase of a two-component regulator system |
| LA1039 | 7.23 | 28.57 | 0.58 | 0.03 | - | CYT |  | hypothetical protein |
| LA1040 | 4.00 | 25.56 | 0.55 | 0.08 | S | NON-CYT |  | Conserved hypothetical lipoprotein |
| LA1044 | 8.00 | 44.85 | 0.55 | 0.02 | M | CYT |  | Conserved hypothetical protein |
| LA1047 | 22.22 | 41.23 | 0.95 | 0.05 | E | CYT | *lysA* | Diaminopimelate decarboxylase |
| LA1080 | 22.72 | 33.82 | 0.71 | 0.04 | E | CYT | *pepN* | Membrane alanyl aminopeptidase |
| LA1082 | 11.52 | 53.65 | 0.85 | 0.06 | L | CYT |  | hypothetical protein with a C-terminal domain related to MutT/nudix family of proteins |
| LA1085 | 2.51 | 20.03 | 0.67 | 0.04 | L | CYT | *uvrD-3* | DNA/RNA helicase UvrD |
| LA1086 | 10.00 | 36.57 | 0.38 | 0.01 | - | NON-CYT |  | TPR-repeat lipoprotein |
| LA1092 | 2.85 | 16.88 | 1.07 | 0.07 | S | CYT |  | Conserved hypothetical protein |
| LA1097 | 6.93 | 31.91 | 1.00 | 0.12 | S | CYT |  | conserved hypothetical protein |
| LA1100 | 24.55 | 44.95 | 0.94 | 0.03 | U | OM |  | TolC-like protein |
| LA1101 | 27.69 | 62.89 | 0.95 | 0.01 | C | CYT | *sucD* | Succinyl-CoA synthetase, alpha subunit |
| LA1102 | 47.76 | 71.54 | 0.89 | 0.03 | C | CYT | *sucC* | Succinyl-CoA synthetase, beta subunit |
| LA1103 | 3.70 | 10.75 | 0.98 | 0.05 | - | CYT |  | hypothetical protein |
| LA1107 | 15.37 | 46.55 | 1.41 | 0.02 | - | CYT |  | Conserved hypothetical protein |
| LA1110 | 29.44 | 49.41 | 0.90 | 0.00 | F | CYT |  | Adenylosuccinate synthase |
| LA1112 | 24.01 | 38.12 | 1.04 | 0.03 | C | CYT |  | Bifunctional glycerol-3-phosphate dehydrogenase /glycerol-3-phosphate acyltransferase |
| LA1118 | 5.70 | 8.23 | 0.17 | 0.02 | - | NON-CYT |  | hypothetical protein |
| LA1119 | 3.52 | 15.17 | 1.25 | 0.02 | F | CYT | *dut* | dUTP diphosphatase |
| LA1126 | 4.00 | 18.58 | 1.01 | 0.12 | E | CYT |  | Phosphoglycerate dehydrogenase |
| LA1135 | 9.71 | 19.54 | 0.56 | 0.06 | - | CYT |  | hypothetical protein |
| LA1137 | 7.40 | 32.35 | 1.40 | 0.07 | S | CYT |  | Transcriptional regulator |
| LA1138 | 11.40 | 18.08 | 0.64 | 0.04 | J | CYT | *miaB-2* | 2-methylthioadenine synthetase |
| LA1140 | 6.00 | 17.56 | 1.15 | 0.23 | E | CYT | *trpD* | Anthranilate phosphoribosyltransferase |
| LA1142 | 10.38 | 34.11 | 1.13 | 0.09 | U | IM | *secD* | Preprotein translocase, SecD subunit |
| LA1144 | 4.05 | 14.16 | 1.23 | 0.04 | H | CYT | *ribD* | Pyrimidine deaminase, riboflavin biosynthesis |
| LA1145 | 4.00 | 15.50 | 1.18 | 0.11 | H | CYT | *ribC* | Riboflavin synthase alpha chain |
| LA1147 | 33.92 | 66.58 | 1.30 | 0.02 | H | CYT | *ribAB* | Bifunctional 3,4-dihydroxy-2-butanone 4-phosphate synthase/GTP cyclohydrolase II |
| LA1152 | 16.85 | 55.26 | 1.36 | 0.06 | - | CYT |  | hypothetical protein |
| LA1155 | 8.56 | 39.10 | 1.58 | 0.13 | P | PER |  | Substrate binding protein of an ABC transporter complex |
| LA1158 | 6.00 | 19.10 | 1.43 | 0.07 | P | CYT |  | ATP-binding protein of an ABC transporter complex |
| LA1162 | 2.00 | 17.47 | 0.83 | 0.22 | - | CYT |  | conserved hypothetical protein |
| LA1173 | 4.00 | 16.82 | 0.57 | 0.05 | I | CYT |  | Acetyl-CoA synthetase |
| LA1174 | 10.37 | 38.18 | 1.32 | 0.08 | E | NON-CYT |  | ATP Binding protein of an ABC transporter complex |
| LA1175 | 2.00 | 12.82 | 0.59 | 0.14 | L | CYT | *mutL* | DNA mismatch repair enzyme (predicted ATPase) |
| LA1179 | 2.00 | 21.04 | 0.65 | 0.24 | L | CYT |  | ATPase related to the helicase subunit of the Holliday junction resolvase |
| LA1182 | 17.16 | 47.41 | 1.21 | 0.04 | E | CYT | *hisC* | Bifunctional histidinol-phosphate/aromatic aminotransferase/cobyric acid decarboxylase |
| LA1184 | 2.00 | 8.68 | 1.12 | 0.38 | T | CYT |  | Adenylate/guanylate cyclase |
| LA1185 | 2.00 | 7.56 | 0.97 | 0.15 | T | CYT |  | Response regulator of a two component complex |
| LA1190 | 11.22 | 26.20 | 1.03 | 0.08 | - | CYT |  | conserved hypothetical protein |
| LA1191 | 4.02 | 16.35 | 0.93 | 0.02 | T | NON-CYT |  | Methyl-accepting chemotaxis protein |
| LA1198 | 13.40 | 52.53 | 0.99 | 0.05 | I | CYT |  | Enoyl-CoA hydratase/isomerase family protein |
| LA1206 | 2.00 | 13.37 | 1.13 | 0.10 | - | CYT |  | hypothetical protein |
| LA1209 | 6.10 | 11.28 | 0.79 | 0.07 | E | NON-CYT | *pepD* | Dipeptidase |
| LA1212 | 2.96 | 18.06 | 1.67 | 0.43 | T | CYT | *uspA* | Universal stress protein UspA |
| LA1220 | 4.02 | 15.41 | 1.08 | 0.11 | R | CYT |  | Metal-dependent hydrolase |
| LA1221 | 4.00 | 7.88 | 0.89 | 0.09 | M | IM |  | Membrane carboxypeptidase/penicillin-binding protein 1 |
| LA1223 | 12.00 | 27.62 | 0.96 | 0.10 | C | CYT | *lpdA-1* | Dihydrolipoamide dehydrogenase |
| LA1224 | 4.00 | 9.46 | 1.00 | 0.17 | C | CYT | *sucA* | Oxoglutarate dehydrogenase (lipoamide), dehydrogenase (E1) component |
| LA1231 | 4.00 | 16.09 | 1.87 | 0.29 | O | CYT |  | Heat shock protein htpG |
| LA1239 | 2.50 | 16.85 | 1.10 | 0.06 | E | CYT | *carA* | Carbamoyl-phosphate synthase (glutamine-hydrolyzing), small subunit |
| LA1240 | 33.50 | 32.29 | 1.04 | 0.03 | J | CYT | *thrS* | Threonine--tRNA ligase |
| LA1242 | 3.97 | 25.70 | 1.14 | 0.03 | J | CYT | *infC* | Translation initiation factor 3 (IF-3) |
| LA1244 | 4.00 | 24.79 | 0.78 | 0.05 | J | CYT | *rplT* | 50S Ribosomal protein L20 |
| LA1245 | 6.38 | 19.34 | 1.60 | 0.35 | - | CYT |  | Hypothetical protein |
| LA1247 | 6.02 | 51.14 | 1.00 | 0.03 | H | CYT |  | 5-formyltetrahydrofolate cyclo-ligase |
| LA1250 | 4.64 | 37.20 | 1.12 | 0.13 | T | CYT | *cheW* | Chemotaxis signal transduction protein |
| LA1252 | 16.00 | 49.58 | 1.24 | 0.04 | T | CYT | *cheB* | Chemotaxis response regulator containing a CheY domain |
| LA1254 | 6.96 | 39.85 | 1.11 | 0.06 | S | CYT |  | Conserved hypothetical protein |
| LA1255 | 4.08 | 40.68 | 1.43 | 0.05 | K | CYT |  | Transcriptional regulator |
| LA1256 | 12.63 | 40.22 | 1.00 | 0.04 | E | CYT | *pheA* | Bifunctional prephenate dehydratase/chorismate mutase |
| LA1257 | 2.00 | 7.87 | 1.07 | 0.18 | E | NON-CYT | *tyrA* | Bifunctional prephenate dehydrogenase/chorismate mutase |
| LA1258 | 6.00 | 24.09 | 0.89 | 0.10 | E | CYT | *aroA* | 3-phosphoshikimate 1-carboxyvinyltransferase |
| LA1259 | 4.00 | 25.33 | 0.91 | 0.02 | F | CYT | *cmk* | Cytidylate kinase |
| LA1260 | 54.69 | 60.00 | 0.68 | 0.01 | J | CYT | *rpsA-2* | 30S Ribosomal protein S1 |
| LA1273 | 13.71 | 67.47 | 0.88 | 0.05 | - | CYT |  | Hypothetical protein |
| LA1293 | 21.99 | 42.35 | 0.93 | 0.02 | E | CYT |  | Aspartate/tyrosine/aromatic aminotransferase |
| LA1295 | 8.05 | 24.16 | 0.94 | 0.11 | R | CYT |  | ATPase |
| LA1303 | 10.82 | 22.29 | 0.95 | 0.04 | R | CYT |  | GTPase |
| LA1309 | 2.05 | 11.26 | 0.76 | 0.17 | D | CYT | *smc* | Chromosome segregation ATPase |
| LA1313 | 9.99 | 25.58 | 1.04 | 0.02 | E | CYT | *glnA* | Glutamate--ammonia ligase |
| LA1323 | 46.66 | 50.87 | 1.00 | 0.05 | F | CYT | *purL* | Phosphoribosylformylglycinamidine synthase |
| LA1325 | 42.40 | 32.71 | 0.99 | 0.03 | J | CYT | *ileS* | Isoleucine--tRNA ligase |
| LA1326 | 11.06 | 34.15 | 1.12 | 0.02 | - | NON-CYT |  | hypothetical lipoprotein |
| LA1327 | 8.00 | 44.78 | 1.20 | 0.04 | T | CYT |  | Anti-sigma factor antagonist |
| LA1328 | 26.44 | 50.33 | 1.37 | 0.01 | - | CYT |  | Hypothetical protein |
| LA1329 | 10.56 | 37.66 | 1.32 | 0.13 | R | CYT |  | Amidohydrolase |
| LA1332 | 8.02 | 38.75 | 1.79 | 0.21 | R | NON-CYT |  | Ankyrin-repeat protein |
| LA1351 | 11.97 | 51.46 | 0.99 | 0.20 | M | NON-CYT | *capA* | Poly-gamma-glutamate biosynthesis protein |
| LA1374 | 2.11 | 15.44 | 1.48 | 0.25 | R | CYT |  | Pirin-related protein |
| LA1378 | 36.36 | 50.00 | 0.85 | 0.01 | T | IM |  | Membrane GTPase |
| LA1384 | 6.00 | 26.14 | 1.18 | 0.12 | - | NON-CYT |  | hypothetical lipoprotein |
| LA1388 | 20.52 | 34.05 | 0.98 | 0.06 | J | CYT | *glyRS* | Glycine--tRNA ligase |
| LA1391 | 6.68 | 30.00 | 0.69 | 0.05 | J | CYT | *prmA* | Ribosomal protein L11 methylase |
| LA1392 | 20.63 | 53.05 | 0.82 | 0.02 | G | CYT |  | Sugar kinase |
| LA1396 | 4.70 | 28.13 | 0.65 | 0.13 | - | NON-CYT |  | hypothetical lipoprotein |
| LA1402 | 33.59 | 45.40 | 0.06 | 0.02 | - | NON-CYT |  | Conserved hypothetical protein |
| LA1409 | 34.05 | 50.36 | 0.94 | 0.03 | E | CYT | *glyA* | Glycine hydroxymethyltransferase |
| LA1416 | 6.00 | 45.91 | 1.05 | 0.08 | G | CYT |  | Phosphonomutase |
| LA1419 | 20.83 | 37.04 | 0.96 | 0.02 | T | CYT |  | Adenylate/guanylate cyclase |
| LA1420 | 2.00 | 8.87 | 0.96 | 0.13 | - | CYT |  | Conserved hypothetical protein |
| LA1421 | 6.17 | 34.78 | 0.63 | 0.05 | - | CYT |  | Conserved hypothetical protein |
| LA1422 | 37.67 | 27.47 | 0.99 | 0.04 | R | CYT |  | Signal transduction protein with multiple domains |
| LA1423 | 15.70 | 37.27 | 1.28 | 0.07 | I | CYT | *fabH* | 3-oxoacyl-[acyl-carrier protein] synthase |
| LA1424 | 8.66 | 18.89 | 0.74 | 0.07 | I | IM | *atoAD* | Acetate CoA-transferase |
| LA1427 | 5.40 | 34.13 | 0.88 | 0.08 | Q | CYT |  | Short chain dehydrogenase |
| LA1428 | 2.00 | 5.20 | 1.42 | 0.30 | T | CYT |  | Serine/threonine specific protein phosphatase |
| LA1430 | 4.00 | 23.78 | 0.90 | 0.09 | Q | CYT | *fabB* | 3-oxoacyl-[acyl-carrier protein] synthase |
| LA1437 | 7.68 | 30.87 | 0.99 | 0.05 | G | CYT |  | Transcriptional regulator/sugar kinase |
| LA1438 | 6.00 | 35.75 | 1.44 | 0.13 | V | CYT |  | ATP-binding protein of an ABC transporter complex |
| LA1445 | 2.00 | 11.52 | 0.66 | 0.15 | U | OM |  | TolC related protein |
| LA1446 | 3.14 | 14.25 | 1.16 | 0.35 | M | NON-CYT |  | Efflux pump |
| LA1447 | 2.61 | 12.32 | 1.13 | 0.01 | K | CYT | *lexA* | Repressor lexA |
| LA1449 | 14.00 | 32.68 | 0.76 | 0.05 | M | PER | *prc-2* | C-terminal processing periplasmic-protease-3 |
| LA1450 | 4.11 | 14.50 | 1.30 | 0.10 | O | SEC |  | O-sialoglycoprotein metal-dependent endopeptidase |
| LA1459 | 18.80 | 32.80 | 1.11 | 0.08 | M | NON-CYT | *ugd* | UDP-glucose 6-dehydrogenase |
| LA1463 | 2.00 | 24.69 | 1.28 | 0.33 | F | CYT | *purE* | Phosphoribosylaminoimidazole carboxylase, catalytic subunit |
| LA1467 | 4.01 | 16.07 | 0.67 | 0.06 | - | NON-CYT |  | hypothetical lipoprotein |
| LA1471 | 10.00 | 13.35 | 1.25 | 0.10 | C | IM |  | Inorganic pyrophosphatase |
| LA1476 | 6.08 | 22.44 | 1.05 | 0.03 | R | CYT |  | Dehalogenase-like hydrolase |
| LA1480 | 6.50 | 28.38 | 1.49 | 0.35 | R | CYT |  | PLP dependent enzyme |
| LA1483 | 16.62 | 39.49 | 1.11 | 0.01 | T | CYT |  | Response regulator, GGDEF family |
| LA1488 | 15.59 | 15.92 | 1.11 | 0.04 | C | IM |  | Dehydrogenase |
| LA1495 | 11.30 | 24.77 | 0.90 | 0.08 | - | NON-CYT |  | hypothetical protein |
| LA1507 | 23.70 | 49.41 | 1.35 | 0.10 | M | NON-CYT |  | OMA87 related protein |
| LA1508 | 6.74 | 25.99 | 1.38 | 0.17 | - | CYT |  | hypothetical protein |
| LA1510 | 4.00 | 21.72 | 1.38 | 0.11 | M | NON-CYT |  | OMA87 related protein |
| LA1511 | 4.98 | 20.86 | 0.88 | 0.12 | H | NON-CYT | *nadB* | L-aspartate oxidase |
| LA1512 | 29.03 | 37.69 | 1.22 | 0.01 | - | CYT |  | hypothetical protein |
| LA1513 | 5.29 | 18.80 | 1.36 | 0.10 | M | NON-CYT | *prc-1* | Periplasmic protease |
| LA1514 | 2.00 | 9.14 | 0.85 | 0.18 | H | CYT | *pdxJ* | Pyridoxal phosphate biosynthesis protein |
| LA1518 | 9.22 | 37.39 | 1.23 | 0.06 | M | CYT | *manC* | Mannose-1-phosphate guanylyltransferase (GDP) |
| LA1523 | 2.00 | 14.04 | 0.99 | 0.37 | - | NON-CYT |  | Hypothetical lipoprotein |
| LA1526 | 4.00 | 22.60 | 1.69 | 0.07 | T | CYT |  | Signal transduction protein containing cAMP-binding and CBS domains |
| LA1528 | 4.00 | 9.02 | 0.71 | 0.11 | T | CYT |  | Receiver domain of a two-component regulator complex |
| LA1533 | 13.99 | 41.98 | 0.77 | 0.04 | F | CYT |  | Thymidylate synthase |
| LA1537 | 2.00 | 8.75 | 0.71 | 0.18 | - | CYT |  | hypothetical protein |
| LA1542 | 2.00 | 10.42 | 0.74 | 0.12 | - | NON-CYT |  | Conserved hypothetical protein |
| LA1553 | 5.70 | 13.22 | 1.28 | 0.13 | - | IM | *petE* | Plastocyanin |
| LA1563 | 9.59 | 60.00 | 7.32 | 0.43 | O | CYT | *ibpA-2* | Small heat shock protein (molecular chaperone) |
| LA1564 | 5.40 | 25.36 | 6.13 | 0.37 | O | CYT | *ibpA-1* | Small heat shock protein (molecular chaperone) |
| LA1569 | 2.04 | 13.08 | 1.06 | 0.21 | - | NON-CYT |  | Conserved hypothetical lipoprotein |
| LA1572 | 2.00 | 10.27 | 1.23 | 0.05 | - | CYT |  | Conserved hypothetical protein |
| LA1577 | 3.40 | 10.19 | 1.22 | 0.03 | M | CYT |  | Nucleoside-diphosphate-sugar epimerase |
| LA1579 | 2.11 | 8.11 | 1.32 | 0.03 | G | CYT | *gmhA* | Phosphoheptose isomerase |
| LA1580 | 20.35 | 44.98 | 1.06 | 0.03 | M | CYT |  | Nucleoside-diphosphate-sugar epimerase |
| LA1581 | 8.00 | 25.09 | 0.69 | 0.13 | M | CYT |  | Nucleoside-diphosphate-sugar pyrophosphorylase |
| LA1582 | 11.69 | 24.95 | 1.12 | 0.05 | M | CYT |  | ADP-heptose synthase |
| LA1583 | 29.94 | 67.88 | 0.90 | 0.04 | M | CYT | *galE* | UDP-glucose 4-epimerase |
| LA1584 | 7.10 | 18.22 | 0.87 | 0.02 | - | CYT |  | Conserved hypothetical protein |
| LA1585 | 16.82 | 45.83 | 0.97 | 0.06 | G | CYT | *tktN* | Transketolase, N-terminal subunit |
| LA1586 | 20.48 | 47.10 | 1.02 | 0.05 | G | CYT | *tktC* | Transketolase, C-terminal subunit |
| LA1587 | 18.80 | 54.92 | 1.23 | 0.04 | - | CYT |  | Conserved hypothetical protein |
| LA1590 | 3.46 | 17.19 | 0.74 | 0.03 | H | CYT |  | Methylase/methyltransferase |
| LA1592 | 29.92 | 49.62 | 0.92 | 0.02 | R | CYT |  | Methyltransferase |
| LA1593 | 4.40 | 22.86 | 0.93 | 0.07 | - | CYT |  | Conserved hypothetical protein |
| LA1594 | 24.19 | 32.84 | 0.93 | 0.03 | H | CYT |  | Conserved hypothetical protein |
| LA1595 | 16.21 | 26.82 | 0.91 | 0.04 | M | CYT |  | pyridoxal-phosphate-dependent aminotransferase |
| LA1602 | 2.00 | 11.07 | 0.66 | 0.04 | H | CYT |  | Methylase/methyltransferase |
| LA1605 | 5.22 | 23.68 | 0.92 | 0.16 | M | CYT | *neuA* | N-acylneuraminate cytidylyltransferase |
| LA1606 | 9.02 | 25.45 | 1.10 | 0.03 | M | CYT |  | Nucleoside-diphosphate-sugar epimerase |
| LA1607 | 4.17 | 19.12 | 1.10 | 0.07 | M | CYT |  | Aminotransferase |
| LA1610 | 5.74 | 19.38 | 0.79 | 0.05 | M | CYT | *wecB* | UDP-N-acetylglucosamine 2-epimerase |
| LA1611 | 6.00 | 21.08 | 1.18 | 0.17 | M | CYT |  | Nucleoside-diphosphate-sugar pyrophosphorylase |
| LA1613 | 18.70 | 30.68 | 0.72 | 0.01 | M | CYT |  | N-acetyl neuraminic (sialic) acid synthetase and isomerase/epimerase |
| LA1614 | 13.78 | 27.16 | 0.84 | 0.02 | M | CYT |  | Aminotransferase |
| LA1615 | 4.00 | 28.23 | 0.63 | 0.05 | M | CYT | *neuA* | N-acylneuraminate cytidylyltransferase |
| LA1616 | 18.42 | 41.41 | 0.96 | 0.02 | C | CYT |  | Alcohol dehyodrogenase |
| LA1617 | 19.12 | 45.85 | 1.09 | 0.03 | R | CYT |  | Fe-S oxidoreductase |
| LA1619 | 19.39 | 28.90 | 0.28 | 0.00 | O | CYT |  | Carbamoyl transferase |
| LA1625 | 13.03 | 35.06 | 0.83 | 0.01 | M | CYT | *kdsB-2* | 3-deoxy-manno-octulosonate cytidylyltransferase |
| LA1626 | 6.83 | 22.81 | 1.13 | 0.15 | R | CYT |  | Oxidoreductase family protein |
| LA1628 | 9.40 | 22.19 | 1.03 | 0.04 | H | CYT |  | Methylase/methyltransferase |
| LA1629 | 19.64 | 37.95 | 1.01 | 0.01 | H | CYT |  | Dehydrogenase |
| LA1632 | 2.02 | 8.82 | 0.90 | 0.07 | M | CYT |  | Nucleoside-diphosphate-sugar epimerase |
| LA1633 | 4.96 | 50.67 | 1.17 | 0.17 | M | CYT |  | RmlC-related protein |
| LA1638 | 4.00 | 20.21 | 1.31 | 0.19 | G | CYT |  | Polysaccharide deacetylase |
| LA1643 | 10.96 | 35.76 | 1.37 | 0.05 | M | CYT |  | N-acetyl glucosamine/N-acetyl galactosamine epimerase |
| LA1644 | 7.19 | 22.13 | 0.93 | 0.02 | M | CYT |  | NDP-sugar dehydratase or epimerase |
| LA1654 | 4.00 | 33.81 | 0.88 | 0.07 | - | CYT |  | Sugar isomerase |
| LA1655 | 9.87 | 50.69 | 0.96 | 0.10 | M | CYT |  | Sugar pyridoxal-phosphate-dependent aminotransferase |
| LA1659 | 8.00 | 24.73 | 1.14 | 0.04 | M | CYT | *rmlC* | dTDP-4-dehydrorhamnose 3,5-epimerase |
| LA1660 | 4.00 | 9.80 | 1.16 | 0.14 | M | CYT | *rmlD* | dTDP-4-dehydrorhamnose reductase |
| LA1661 | 6.03 | 25.50 | 0.90 | 0.04 | M | CYT | *rmlB* | dTDP-glucose 4,6-dehydratase |
| LA1662 | 10.00 | 26.87 | 1.08 | 0.06 | M | CYT | *rmlA* | Glucose-1-phosphate thymidylyltransferase |
| LA1671 | 2.00 | 12.15 | 0.50 | 0.09 | R | CYT |  | Ankyrin repeat protein |
| LA1675 | 4.00 | 20.88 | 0.92 | 0.06 | J | CYT | *rpsF* | 30S Ribosomal protein S6 |
| LA1676 | 6.01 | 47.33 | 0.92 | 0.18 | L | CYT | *ssb* | Single-stranded DNA-binding protein |
| LA1677 | 3.05 | 38.27 | 0.90 | 0.03 | J | CYT | *rpsR* | 30S Ribosomal protein S18 |
| LA1678 | 6.00 | 37.58 | 1.44 | 0.15 | J | CYT | *rplI* | 50S Ribosomal protein L9 |
| LA1680 | 44.22 | 50.58 | 0.95 | 0.03 | J | CYT | *aspS* | Aspartate--tRNA ligase |
| LA1688 | 33.73 | 41.98 | 0.91 | 0.01 | J | CYT | *argS* | Arginine--tRNA ligase |
| LA1689 | 8.00 | 16.03 | 0.65 | 0.06 | R | CYT |  | CinA-related protein |
| LA1692 | 7.52 | 17.27 | 0.82 | 0.11 | T | CYT |  | Receiver component of a two-component response regulator |
| LA1696 | 8.06 | 30.00 | 0.99 | 0.02 | G | CYT | *tpiA* | Triosephosphate isomerase |
| LA1703 | 34.84 | 55.05 | 1.01 | 0.02 | G | CYT | *pgk* | Phosphoglycerate kinase |
| LA1704 | 26.31 | 59.40 | 1.07 | 0.01 | G | CYT | *gapA* | Glyceraldehyde 3-phosphate dehydrogenase (phosphorylating) |
| LA1719 | 43.69 | 87.06 | 1.31 | 0.03 | E | CYT | *cysK-2* | Cysteine synthase |
| LA1726 | 13.12 | 33.06 | 0.69 | 0.03 | T | CYT |  | Signal transduction protein |
| LA1728 | 4.00 | 10.99 | 0.64 | 0.07 | - | NON-CYT |  | hypothetical protein |
| LA1730 | 3.74 | 44.63 | 1.22 | 0.10 | - | CYT |  | hypothetical protein |
| LA1793 | 14.21 | 39.86 | 0.88 | 0.12 | S | CYT |  | Transposase, YhgA-like |
| LA1830 | 2.00 | 34.21 | 0.81 | 0.11 | S | CYT |  | Transposase, YhgA-like |
| LA1841 | 17.22 | 58.44 | 0.55 | 0.03 | - | CYT |  | hypothetical protein |
| LA1853 | 10.00 | 36.99 | 1.08 | 0.11 | S | CYT |  | Conserved hypothetical protein |
| LA1859 | 14.66 | 31.81 | 3.76 | 0.06 | P | CYT | *katE* | Catalase |
| LA1862 | 13.52 | 37.25 | 1.16 | 0.16 | J | CYT | *spoU* | tRNA (guanosine-2'-O-)-methyltransferase |
| LA1863 | 22.16 | 43.31 | 0.97 | 0.03 | J | CYT | *cysS* | Cysteine--tRNA ligase |
| LA1864 | 16.42 | 43.30 | 0.88 | 0.05 | Q | CYT |  | Acetyl esterase |
| LA1865 | 13.40 | 48.24 | 1.14 | 0.09 | H | CYT | *folD* | Bifunctional folate pathway enzyme |
| LA1866 | 24.19 | 48.05 | 1.33 | 0.02 | J | CYT | *asnS* | Asparagine--tRNA ligase |
| LA1867 | 4.62 | 11.79 | 1.12 | 0.64 | - | CYT |  | Hypothetical protein |
| LA1875 | 4.18 | 17.48 | 1.07 | 0.14 | L | CYT |  | DNA helicase |
| LA1879 | 36.71 | 47.79 | 2.84 | 0.09 | O | CYT | *clpA-1* | Endopeptidase Clp, ATP-dependent proteolytic subunit |
| LA1883 | 15.15 | 26.14 | 0.84 | 0.09 | - | NON-CYT |  | Hypothetical lipoprotein |
| LA1888 | 22.70 | 30.83 | 0.89 | 0.07 | M | IM | *lepA* | GTPase |
| LA1890 | 2.00 | 7.88 | 1.84 | 0.24 | E | CYT |  | Conserved hypothetical protein |
| LA1897 | 8.05 | 16.01 | 1.07 | 0.18 | C | PER | *sdhA* | Succinate dehydrogenase/fumarate reductase subunit A |
| LA1910 | 2.74 | 15.84 | 1.26 | 0.05 | - | CYT |  | hypothetical protein |
| LA1911 | 15.29 | 44.23 | 1.06 | 0.04 | H | CYT |  | Dehydrogenase |
| LA1912 | 2.32 | 11.24 | 0.49 | 0.18 | - | NON-CYT |  | Hypothetical lipoprotein |
| LA1915 | 7.22 | 16.26 | 0.55 | 0.06 | - | OM |  | TPR-repeat-containing protein |
| LA1929 | 37.97 | 41.03 | 0.99 | 0.00 | T | CYT |  | CAP family transcription factor |
| LA1930 | 7.22 | 28.46 | 0.69 | 0.14 | I | CYT | *caiA-2* | Acyl-CoA dehydrogenase |
| LA1934 | 4.89 | 39.85 | 1.06 | 0.16 | - | CYT |  | conserved hypothetical protein |
| LA1935 | 14.00 | 44.21 | 0.92 | 0.03 | S | CYT |  | conserved hypothetical protein |
| LA1939 | 16.97 | 54.09 | 1.32 | 0.02 | - | NON-CYT |  | hypothetical lipoprotein |
| LA1947 | 7.00 | 20.90 | 1.13 | 0.13 | C | NON-CYT |  | Conserved hypothetical protein |
| LA1948 | 4.00 | 17.90 | 1.21 | 0.12 | T | CYT |  | Receiver protein of a two-component response regulator |
| LA1951 | 14.54 | 31.71 | 1.00 | 0.03 | G | CYT | *eno* | Enolase |
| LA1953 | 12.54 | 48.73 | 1.49 | 0.08 | O | CYT | *clpP-2* | Protease subunit of an ATP-dependent Clp protease |
| LA1954 | 2.01 | 19.49 | 1.03 | 0.10 | - | CYT |  | hypothetical protein |
| LA1956 | 2.05 | 24.32 | 1.95 | 0.18 | - | CYT |  | Conserved hypothetical protein |
| LA1957 | 4.00 | 13.61 | 2.58 | 0.28 | - | NON-CYT |  | Hypothetical protein |
| LA1959 | 2.19 | 14.06 | 0.99 | 0.02 | F | CYT | *guaA-1* | Glutamine amidotransferase |
| LA1960 | 45.35 | 39.89 | 0.91 | 0.01 | U | CYT | *secA* | Preprotein translocase, SecA subunit |
| LA1961 | 2.00 | 7.79 | 1.23 | 0.33 | - | NON-CYT |  | hypothetical protein |
| LA1970 | 5.56 | 28.24 | 1.06 | 0.23 | J | CYT |  | Poly A polymerase family of protein |
| LA1975 | 10.00 | 35.37 | 0.82 | 0.01 | R | CYT |  | Short chain dehydrogenase |
| LA1983 | 2.00 | 2.59 | 0.27 | 0.13 | T | CYT |  | Signal transduction protein |
| LA1985 | 7.07 | 22.43 | 1.03 | 0.30 | - | CYT |  | hypothetical protein |
| LA1995 | 37.06 | 62.63 | 0.87 | 0.03 | J | CYT | *lysU* | Lysine--tRNA ligase |
| LA1996 | 2.00 | 13.43 | 1.36 | 0.16 | - | IM |  | hypothetical protein |
| LA2017 | 14.00 | 49.47 | 0.39 | 0.02 | N | PER | *flaB1* | Endoflagellar filament core protein |
| LA2019 | 4.39 | 33.57 | 0.57 | 0.03 | N | PER | *flaB-2* | Endoflagellar filament core protein |
| LA2020 | 16.32 | 35.33 | 0.25 | 0.01 | - | NON-CYT |  | hypothetical protein |
| LA2024 | 58.59 | 80.83 | 0.86 | 0.01 | - | OM |  | conserved hypothetical lipoprotein |
| LA2025 | 16.00 | 29.29 | 1.25 | 0.06 | S | OM |  | conserved hypothetical protein |
| LA2030 | 2.00 | 12.50 | 0.96 | 0.02 | R | NON-CYT |  | Zinc dependent protease/lipoprotein |
| LA2036 | 6.03 | 21.17 | 0.95 | 0.06 | - | NON-CYT |  | Hypothetical lipoprotein |
| LA2042 | 8.32 | 38.28 | 1.28 | 0.09 | T | CYT | *cheR* | Methyltransferase of chemotaxis protein |
| LA2045 | 2.00 | 10.06 | 0.47 | 0.15 | M | CYT | *mraW* | S-adenosylmethionine-dependent methyltransferase |
| LA2047 | 20.30 | 31.03 | 0.81 | 0.06 | M | CYT | *murE* | UDP-N-acetylmuramoylalanyl-D-glutamate--2,6-diaminopimelate ligase |
| LA2051 | 10.24 | 24.90 | 1.07 | 0.06 | M | PER | *murC-1* | UDP-N-acetylmuramate--L-alanine ligase |
| LA2058 | 11.10 | 36.18 | 0.94 | 0.02 | M | CYT | *ddlA* | D-alanine--D-alanine ligase |
| LA2062 | 22.87 | 38.94 | 1.32 | 0.03 | E | CYT | *metY* | O-acetylhomoserine aminocarboxypropyltransferase |
| LA2066 | 9.58 | 44.93 | 0.31 | 0.04 | - | NON-CYT |  | Hypothetical protein |
| LA2068 | 2.00 | 15.89 | 1.03 | 0.17 | O | NON-CYT |  | AAA family ATPase |
| LA2069 | 2.00 | 13.48 | 0.76 | 0.05 | N | CYT | *fliN-2* | Endoflagellar motor switch protein |
| LA2076 | 4.22 | 22.77 | 1.23 | 0.17 | E | CYT | *argH* | Argininosuccinate lyase |
| LA2081 | 4.00 | 19.35 | 0.90 | 0.04 | N | CYT | *fliM* | Endoflagellar motor switch protein |
| LA2085 | 9.32 | 51.11 | 1.21 | 0.03 | R | CYT |  | GTP cyclohydrolase-1 related protein |
| LA2087 | 18.02 | 37.65 | 0.54 | 0.03 | F | CYT | *guaA-2* | GMP synthase (glutamine-hydrolyzing) |
| LA2095 | 21.62 | 42.80 | 0.96 | 0.01 | E | CYT | *leuC* | 3-isopropylmalate dehydratase, large subunit |
| LA2096 | 16.51 | 62.14 | 0.93 | 0.03 | E | CYT | *leuD* | 3-isopropylmalate dehydratase, small subunit |
| LA2098 | 6.47 | 17.51 | 0.91 | 0.14 | L | CYT |  | DNA and RNA helicase subunit |
| LA2104 | 20.01 | 27.21 | 0.97 | 0.06 | O | CYT | *clpA-2* | ATP-dependent clp protease ATP-binding subunit |
| LA2109 | 4.85 | 42.99 | 1.24 | 0.22 | O | CYT |  | Glutaredoxin-related protein |
| LA2119 | 2.05 | 15.73 | 1.06 | 0.13 | C | CYT | *glpK-1* | Glycerol kinase |
| LA2126 | 8.21 | 41.46 | 1.03 | 0.04 | O | NON-CYT | *sppA-2* | Periplasmic serine protease (ClpP class) |
| LA2129 | 41.57 | 58.52 | 0.88 | 0.02 | L | CYT |  | DNA or RNA helicase of superfamily II |
| LA2130 | 21.51 | 53.96 | 1.10 | 0.05 | E | CYT |  | Aspartate/tyrosine/aromatic aminotransferase |
| LA2132 | 4.50 | 22.74 | 1.09 | 0.08 | R | CYT |  | Hydrolase or acyltransferase |
| LA2136 | 2.00 | 23.00 | 2.04 | 0.19 | - | NON-CYT |  | hypothetical protein |
| LA2138 | 8.36 | 29.02 | 0.73 | 0.13 | O | CYT |  | Oxidoreductase |
| LA2139 | 38.08 | 69.33 | 1.00 | 0.01 | C | CYT | *mdh* | Malate dehydrogenase |
| LA2140 | 4.53 | 17.55 | 0.85 | 0.09 | H | CYT | *bioF* | 8-amino-7-oxononanoate synthase |
| LA2141 | 2.00 | 16.29 | 0.78 | 0.10 | H | CYT | *bioD* | Dethiobiotin synthetase |
| LA2142 | 6.00 | 10.62 | 0.99 | 0.10 | H | CYT | *bioA* | Adenosylmethionine-8-amino-7-oxononanoate aminotransferase |
| LA2143 | 12.06 | 36.59 | 0.79 | 0.02 | H | CYT | *bioB* | Biotin synthase |
| LA2144 | 8.38 | 34.27 | 0.95 | 0.06 | E | NON-CYT |  | Lipase/phospolipase |
| LA2145 | 2.06 | 9.20 | 0.52 | 0.02 | E | CYT | *serB* | Phosphoserine phosphatase |
| LA2146 | 3.74 | 22.53 | 1.34 | 0.15 | L | CYT | *mutS* | DNA mismatch repair ATPase |
| LA2152 | 20.69 | 44.41 | 1.05 | 0.03 | E | CYT | *leuB-1* | 3-isopropylmalate dehydrogenase |
| LA2153 | 20.78 | 48.28 | 0.98 | 0.05 | E | CYT | *argD* | Acetylornithine aminotransferase |
| LA2154 | 15.04 | 30.32 | 1.28 | 0.12 | G | CYT | *manB-2* | Phosphomannomutase |
| LA2162 | 2.36 | 29.79 | 0.83 | 0.04 | J | CYT | *rpmB* | 50S Ribosomal protein L28 |
| LA2163 | 2.77 | 15.95 | 1.03 | 0.12 | L | CYT |  | Endonuclease III related protein |
| LA2174 | 14.43 | 53.54 | 0.99 | 0.01 | - | CYT |  | Conserved hypothetical protein |
| LA2178 | 18.00 | 38.76 | 1.10 | 0.09 | E | CYT | *argC* | N-acetyl-gamma-glutamyl-phosphate reductase |
| LA2179 | 12.00 | 38.25 | 0.91 | 0.06 | L | CYT | *recA* | RecA recombinase |
| LA2184 | 14.13 | 30.80 | 0.72 | 0.05 | R | UNK |  | Zn-dependent protease |
| LA2185 | 14.70 | 41.67 | 0.69 | 0.02 | R | UNK |  | Zn-dependent protease |
| LA2186 | 22.80 | 22.13 | 0.58 | 0.06 | R | OM |  | Peptidase inhibitor homologue |
| LA2188 | 2.16 | 18.11 | 1.11 | 0.22 | R | CYT |  | ATPase |
| LA2194 | 7.73 | 40.38 | 0.29 | 0.01 | O | CYT | *slpA* | Peptidylprolyl isomerase |
| LA2197 | 18.08 | 35.87 | 0.66 | 0.01 | C | CYT | *fadH* | 2,4-dienoyl-CoA reductase [NADPH] |
| LA2202 | 10.35 | 20.48 | 0.96 | 0.07 | E | CYT | *leuA-2* | 2-isopropylmalate synthase |
| LA2207 | 2.00 | 4.68 | 1.12 | 0.22 | S | IM |  | Conserved hypothetical protein |
| LA2209 | 2.12 | 9.21 | 0.94 | 0.24 | J | CYT |  | Translation factor, SUA5 family |
| LA2212 | 2.00 | 10.65 | 0.71 | 0.17 | L | CYT | *uvrA* | Excinuclease ABC, subunit A |
| LA2213 | 12.28 | 33.81 | 1.09 | 0.06 | - | CYT |  | Hypothetical protein |
| LA2215 | 14.96 | 35.42 | 1.34 | 0.05 | N | IM |  | Endoflagellar motor protein |
| LA2219 | 5.92 | 18.98 | 0.94 | 0.14 | - | NON-CYT |  | Hypothetical lipoprotein |
| LA2223 | 4.08 | 13.87 | 1.02 | 0.13 | T | CYT |  | Sensor histidine kinase of a two component response regulator |
| LA2226 | 13.79 | 39.47 | 0.87 | 0.02 | G | CYT | *fbp* | Fructose-bisphosphatase |
| LA2231 | 8.36 | 22.58 | 0.88 | 0.22 | L | CYT | *dnaG* | DNA primase |
| LA2232 | 6.72 | 20.85 | 0.78 | 0.06 | K | CYT | *rpoD* | DNA-directed RNA polymerase, sigma subunit (sigma70/sigma32) |
| LA2236 | 8.54 | 29.64 | 1.15 | 0.02 | C | CYT | *glpA-1* | Glycerol-3-phosphate dehydrogenase |
| LA2237 | 11.18 | 23.46 | 1.29 | 0.04 | J | CYT | *tyrS* | Tyrosine--tRNA ligase |
| LA2241 | 8.00 | 31.18 | 0.76 | 0.04 | - | NON-CYT |  | Hypothetical lipoprotein |
| LA2244 | 12.55 | 50.60 | 1.00 | 0.06 | - | NON-CYT |  | Hypothetical protein |
| LA2250 | 10.74 | 25.35 | 0.64 | 0.01 | - | CYT |  | Nuclease S1 |
| LA2260 | 4.00 | 26.04 | 1.29 | 0.05 | K | CYT |  | Transcriptional regulator, AcrR-family |
| LA2265 | 4.75 | 29.02 | 1.27 | 0.09 | I | CYT | *caiD-2* | Enoyl-CoA hydratase/carnithine racemase |
| LA2267 | 10.96 | 14.50 | 0.50 | 0.02 | U | NON-CYT |  | conserved hypothetical protein with tetratricopeptide repeat domains |
| LA2280 | 6.00 | 22.67 | 0.79 | 0.09 | O | CYT | *fliS* | Endoflagellar biosynthesis chaperone |
| LA2283 | 31.81 | 64.19 | 0.67 | 0.03 | F | CYT | *purH* | Bifunctional purine biosynthesis protein |
| LA2286 | 15.05 | 64.95 | 1.18 | 0.08 | G | CYT | *MipB* | Transaldolase |
| LA2289 | 2.28 | 16.09 | 1.08 | 0.18 | - | CYT |  | Hypothetical protein |
| LA2295 | 33.43 | 60.57 | 1.68 | 0.02 | S | IM | *lipL45* | Lipoprotein LipL45 |
| LA2297 | 6.77 | 14.45 | 1.04 | 0.08 | - | CYT |  | Conserved hypothetical protein |
| LA2300 | 4.00 | 19.91 | 0.92 | 0.28 | S | CYT |  | Conserved hypothetical protein |
| LA2302 | 3.59 | 28.96 | 0.99 | 0.16 | S | CYT |  | Conserved hypothetical protein |
| LA2303 | 8.28 | 30.63 | 1.10 | 0.05 | R | CYT |  | Short chain dehydrogenase |
| LA2306 | 9.11 | 25.25 | 1.18 | 0.07 | M | CYT | *lpxC* | UDP-3-O-acyl-N-acetylglucosamine deacetylase |
| LA2309 | 24.16 | 37.00 | 0.81 | 0.04 | I | CYT |  | Long-chain-fatty-acid--CoA ligase |
| LA2312 | 3.70 | 37.58 | 2.17 | 0.06 | O | NON-CYT |  | Thiol-disulfide isomerase or thioredoxin |
| LA2323 | 5.73 | 27.78 | 0.78 | 0.03 | - | CYT |  | Conserved hypothetical protein |
| LA2324 | 13.13 | 34.77 | 1.01 | 0.02 | Q | CYT |  | Dehydrogenase |
| LA2325 | 10.10 | 68.69 | 1.33 | 0.03 | K | CYT |  | Transcriptional regulator, ArsR family |
| LA2329 | 25.61 | 41.10 | 1.07 | 0.04 | J | CYT |  | GTPase, probable translation factor |
| LA2337 | 17.13 | 35.16 | 1.23 | 0.08 | D | CYT | *mrp* | ATPase involved in chromosome partitioning |
| LA2340 | 2.02 | 13.41 | 1.13 | 0.15 | - | CYT |  | hypothetical protein |
| LA2343 | 8.00 | 30.14 | 0.81 | 0.04 | G | CYT |  | Dehydrogenase |
| LA2345 | 2.00 | 6.47 | 0.94 | 0.18 | O | CYT | *hslU* | ATP-dependent protease HslVU (ClpYQ), ATPase subunit |
| LA2350 | 28.12 | 43.99 | 1.37 | 0.03 | E | CYT | *leuA-3* | 2-isopropylmalate/homocitrate synthase |
| LA2352 | 6.46 | 13.10 | 1.04 | 0.05 | - | CYT |  | Hypothetical protein |
| LA2360 | 2.85 | 7.41 | 0.43 | 0.05 | F | CYT | *nrdA* | Ribonucleoside-triphosphate reductase, alpha subunit |
| LA2365 | 5.47 | 22.36 | 1.25 | 0.09 | - | CYT |  | Hypothetical protein |
| LA2366 | 7.40 | 28.57 | 1.42 | 0.17 | - | CYT |  | Hypothetical protein |
| LA2367 | 16.56 | 34.26 | 1.14 | 0.10 | D | CYT |  | Actin-like ATPase involved in cell division |
| LA2374 | 21.53 | 46.86 | 1.17 | 0.06 | U | CYT | *gspE* | Type II secretory pathway ATPase, protein E |
| LA2375 | 17.43 | 38.93 | 0.94 | 0.01 | U | IM | *gspD* | Type II secretory pathway component, protein D |
| LA2387 | 5.58 | 27.54 | 0.89 | 0.04 | J | CYT | *rplS* | 50S Ribosomal protein L19 |
| LA2390 | 4.00 | 52.63 | 1.05 | 0.09 | R | CYT |  | RNA-binding protein |
| LA2396 | 7.78 | 27.30 | 1.27 | 0.05 | J | CYT | *fmt* | Methionyl-tRNA formyltransferase |
| LA2398 | 4.04 | 21.48 | 1.05 | 0.17 | L | CYT | *priA* | Primosomal protein N' |
| LA2400 | 16.07 | 47.10 | 1.33 | 0.15 | T | CYT |  | Response regulator of a two component response regulator |
| LA2403 | 15.60 | 39.88 | 1.16 | 0.03 | T | CYT | *hprK* | HPr Serine kinase |
| LA2404 | 6.32 | 24.74 | 0.98 | 0.16 | K | CYT | *rpoN* | DNA-directed RNA polymerase sigma-54 subunit |
| LA2408 | 15.04 | 58.82 | 1.21 | 0.06 | M | CYT | *kdsA* | 3-deoxy-8-phosphooctulonate synthase |
| LA2409 | 19.13 | 25.46 | 1.01 | 0.04 | F | CYT | *pyrG* | CTP synthase |
| LA2411 | 17.57 | 41.54 | 1.15 | 0.07 | M | CYT |  | ADP-heptose synthase, sugar kinase component |
| LA2413 | 5.10 | 15.60 | 1.50 | 0.11 | M | NON-CYT |  | Cell wall-associated hydrolase |
| LA2418 | 4.00 | 40.78 | 0.39 | 0.07 | N | PER | *flaB-1* | Endoflagellar filament core protein |
| LA2420 | 8.01 | 24.55 | 0.86 | 0.17 | I | CYT | *lytB* | Penicillin tolerance protein |
| LA2434 | 2.00 | 26.61 | 1.14 | 0.21 | T | CYT |  | Antagonist of anti-sigma factor |
| LA2436 | 4.00 | 27.45 | 0.72 | 0.08 | - | CYT |  | Hypothetical protein |
| LA2438 | 11.81 | 68.54 | 1.26 | 0.05 | J | CYT | *def* | N-formylmethionyl-tRNA deformylase |
| LA2439 | 2.00 | 7.69 | 0.71 | 0.15 | V | IM |  | Efflux pump, AcrB family |
| LA2457 | 6.88 | 27.38 | 0.92 | 0.10 | J | CYT | *map* | Methionyl aminopeptidase |
| LA2466 | 4.46 | 15.20 | 1.03 | 0.07 | O | IM |  | Methyltransferase |
| LA2468 | 12.80 | 49.15 | 1.32 | 0.09 | - | NON-CYT |  | Hypothetical protein |
| LA2469 | 2.41 | 25.32 | 1.19 | 0.06 | N | CYT | *cheX* | Inhibitor of MCP methylation |
| LA2478 | 2.00 | 7.58 | 1.26 | 0.16 | - | NON-CYT |  | Hypothetical lipoprotein |
| LA2483 | 2.13 | 13.42 | 1.67 | 0.04 | L | CYT | *xerD* | Site-specific recombinase XerD |
| LA2505 | 8.00 | 27.10 | 0.46 | 0.05 | R | CYT |  | Hydrolase or acyltransferase |
| LA2506 | 6.02 | 22.27 | 1.07 | 0.15 | E | CYT | *hisF-1* | Imidazoleglycerol-phosphate synthase |
| LA2507 | 44.35 | 65.91 | 1.08 | 0.02 | J | CYT |  | Amidase |
| LA2512 | 10.02 | 29.05 | 1.38 | 0.05 | - | IM | *lipL31* | Cytoplasmic membrane lipoprotein, LipL31 |
| LA2513 | 22.00 | 31.20 | 0.76 | 0.03 | L | CYT | *mfd* | Transcription-repair coupling factor |
| LA2514 | 6.00 | 30.18 | 0.81 | 0.11 | H | CYT | *panC* | Pantoate--beta-alanine ligase |
| LA2515 | 8.24 | 33.26 | 2.24 | 0.26 | E | CYT | *hisD* | Histidinol dehydrogenase |
| LA2526 | 4.13 | 8.11 | 1.04 | 0.07 | R | CYT |  | Hydrolase or acyltransferase |
| LA2528 | 6.00 | 21.21 | 0.77 | 0.12 | T | CYT |  | GGDEF domain receiver component of a two-component response regulator |
| LA2535 | 15.17 | 87.50 | 0.44 | 0.03 | O | CYT | *ppiB-1* | Peptidylprolyl isomerase |
| LA2536 | 2.00 | 7.50 | 1.12 | 0.44 | T | IM |  | Adenylate cyclase related protein |
| LA2538 | 2.29 | 25.13 | 1.38 | 0.11 | - | NON-CYT |  | Hypothetical protein |
| LA2539 | 6.00 | 18.46 | 0.78 | 0.19 | R | CYT |  | ATP-binding protein of an ABC transporter complex |
| LA2558 | 24.19 | 59.05 | 0.78 | 0.03 | O | CYT | *clpX* | ATPase subunit of endopeptidase Clp |
| LA2559 | 4.96 | 18.18 | 0.90 | 0.06 | O | CYT | *clpP-1* | Protease subunit of endopeptidase Clp |
| LA2560 | 30.24 | 47.45 | 0.87 | 0.03 | O | CYT |  | FKBP-type peptidyl-prolyl cis-trans isomerase (trigger factor) |
| LA2561 | 25.40 | 37.76 | 0.70 | 0.01 | G | NON-CYT |  | Beta-glucosidase-related glycosidase |
| LA2563 | 2.00 | 17.27 | 1.24 | 0.26 | H | CYT |  | Fe-S oxidoreductase |
| LA2570 | 2.93 | 18.45 | 0.36 | 0.10 | E | CYT |  | Thiamine pyrophosphate-requiring enzyme |
| LA2572 | 26.01 | 44.17 | 0.96 | 0.03 | U | CYT |  | Tetratricopeptide repeat-containing protein |
| LA2576 | 13.84 | 25.79 | 1.16 | 0.03 | - | CYT |  | Conserved hypothetical protein |
| LA2577 | 6.00 | 25.12 | 0.81 | 0.07 | R | CYT |  | Unusual protein kinase |
| LA2581 | 6.58 | 16.87 | 1.11 | 0.33 | M | CYT | *neuB-1* | N-acetyl neuraminic (sialic) acid synthetase |
| LA2582 | 7.18 | 20.00 | 0.84 | 0.09 | M | NON-CYT |  | Metallopeptidase |
| LA2584 | 2.00 | 8.78 | 1.08 | 0.06 | S | CYT |  | Conserved hypothetical protein |
| LA2598 | 2.00 | 12.44 | 1.34 | 0.11 | F | CYT | *gmk* | Guanylate kinase |
| LA2602 | 5.92 | 16.34 | 0.78 | 0.06 | L | CYT |  | Polymerase |
| LA2603 | 13.12 | 39.85 | 1.03 | 0.02 | K | CYT |  | RNA polymerase sigma subunit |
| LA2613 | 14.66 | 50.00 | 1.16 | 0.08 | N | CYT | *fliN-1* | Endoflagellar motor switch protein |
| LA2615 | 2.00 | 43.52 | 1.02 | 0.23 | S | CYT |  | Conserved hypothetical protein |
| LA2621 | 25.37 | 67.82 | 0.83 | 0.03 | R | CYT |  | Short chain dehydrogenase |
| LA2623 | 9.72 | 41.74 | 1.40 | 0.09 | O | CYT | *gst-2* | Glutathione transferase |
| LA2625 | 15.49 | 31.49 | 0.78 | 0.04 | E | CYT |  | Zn-dependent oligopeptidase |
| LA2627 | 29.35 | 65.82 | 0.90 | 0.02 | I | CYT | *maoC* | Acyl dehydratase |
| LA2632 | 11.38 | 30.63 | 1.16 | 0.05 | G | CYT | *tktC* | Transketolase, C-terminal subunit |
| LA2633 | 46.33 | 55.96 | 1.06 | 0.01 | H | CYT | *metK* | Methionine adenosyltransferase |
| LA2637 | 85.86 | 96.69 | 0.82 | 0.02 | - | OM | *lipL32* | Major outer membrane protein (MOMP), LipL32 lipoprotein |
| LA2655 | 11.15 | 28.75 | 2.05 | 0.28 | O | CYT | *groEL* | GroEL chaperone |
| LA2674 | 2.31 | 16.54 | 0.89 | 0.12 | R | CYT |  | Metal-dependent amidase/aminoacylase/carboxypeptidase |
| LA2689 | 4.00 | 20.43 | 0.89 | 0.09 | I | CYT | *paaJ-3* | Acetyl-CoA acetyltransferase |
| LA2690 | 4.24 | 37.42 | 0.73 | 0.05 | P | CYT | *bfr* | Bacterioferritin (cytochrome b1) |
| LA2691 | 2.18 | 9.38 | 0.58 | 0.03 | L | CYT |  | N6-adenine-specific DNA methylase |
| LA2699 | 2.00 | 12.30 | 1.52 | 0.27 | M | CYT |  | D-alanine--D-alanine ligase |
| LA2702 | 14.04 | 47.77 | 0.97 | 0.07 | R | CYT |  | Phosphatase |
| LA2705 | 3.70 | 16.39 | 1.07 | 0.07 | - | NON-CYT |  | hypothetical lipoprotein |
| LA2706 | 8.00 | 20.16 | 1.08 | 0.14 | H | CYT | *thiH* | Thiamine biosynthesis enzyme |
| LA2712 | 7.73 | 15.37 | 1.09 | 0.06 | C | IM |  | Fe-S oxidoreductase |
| LA2726 | 6.22 | 20.75 | 1.14 | 0.13 | S | CYT |  | Conserved hypothetical protein |
| LA2727 | 4.01 | 25.00 | 1.23 | 0.11 | R | CYT |  | Dioxygenase |
| LA2733 | 23.38 | 45.98 | 0.82 | 0.02 | J | CYT | *prfC* | Peptide chain release factor 3 |
| LA2739 | 12.02 | 40.61 | 1.16 | 0.01 | S | CYT |  | Conserved hypothetical protein |
| LA2749 | 6.00 | 31.62 | 0.90 | 0.22 | H | CYT |  | 6-pyruvoyl-tetrahydropterin synthase |
| LA2759 | 43.67 | 67.94 | 0.74 | 0.01 | D | CYT | *mreB* | Actin-like ATPase involved in cell morphogenesis |
| LA2773 | 3.75 | 3.11 | 1.16 | 0.09 | - | CYT |  | Conserved hypothetical protein |
| LA2776 | 21.61 | 42.40 | 1.05 | 0.02 | C | CYT | *atpD* | Beta subunit of the H(+)-transporting two-sector ATPase, F1 sector |
| LA2779 | 17.49 | 33.40 | 0.88 | 0.06 | C | CYT | *atpA* | Alpha subunit of the H(+)-transporting two-sector ATPase, F1 sector |
| LA2788 | 16.88 | 49.55 | 1.07 | 0.06 | U | IM | *lepB-2* | Signal peptidase I |
| LA2790 | 2.00 | 16.94 | 2.19 | 0.28 | K | CYT |  | Transcriptional regulator, AcrR-family |
| LA2800 | 14.01 | 27.99 | 1.50 | 0.10 | - | NON-CYT |  | Conserved hypothetical protein |
| LA2808 | 2.00 | 10.40 | 0.96 | 0.08 | O | CYT | *sufB-1* | ABC-type transport system involved in Fe-S cluster assembly, permease component |
| LA2809 | 13.62 | 45.08 | 1.60 | 0.03 | O | CYT | *ahpC* | Peroxiredoxin |
| LA2820 | 2.00 | 8.14 | 0.67 | 0.10 | - | NON-CYT |  | Conserved hypothetical protein |
| LA2821 | 12.29 | 82.09 | 1.50 | 0.07 | - | CYT |  | Conserved hypothetical protein |
| LA2826 | 2.00 | 9.41 | 1.43 | 0.27 | - | CYT |  | hypothetical protein |
| LA2827 | 2.00 | 10.40 | 1.33 | 0.24 | T | CYT |  | Response regulator |
| LA2834 | 28.41 | 41.13 | 0.87 | 0.02 | T | CYT |  | Adenylate/guanylate cyclase |
| LA2835 | 4.03 | 33.58 | 0.89 | 0.03 | - | CYT |  | Conserved hypothetical protein |
| LA2836 | 13.55 | 65.16 | 0.92 | 0.05 | - | CYT |  | Conserved hypothetical protein |
| LA2841 | 18.07 | 42.12 | 1.15 | 0.04 | G | CYT | *citE* | Citrate lyase, beta subunit |
| LA2857 | 13.15 | 34.48 | 0.81 | 0.02 | R | NON-CYT |  | Hydrolase or acyltransferase |
| LA2866 | 3.40 | 12.34 | 0.91 | 0.13 | - | CYT |  | hypothetical protein |
| LA2868 | 21.52 | 45.05 | 1.06 | 0.05 | J | CYT |  | Translation initiation factor 2B |
| LA2882 | 10.44 | 48.21 | 0.78 | 0.01 | - | CYT |  | hypothetical protein |
| LA2892 | 4.29 | 14.63 | 1.18 | 0.03 | - | NON-CYT |  | hypothetical protein |
| LA2894 | 5.15 | 22.66 | 0.98 | 0.06 | K | CYT |  | Transcriptional regulator |
| LA2903 | 2.00 | 12.26 | 1.01 | 0.15 | L | CYT | *ruvA* | Holliday junction specific DNA binding protein |
| LA2918 | 16.31 | 32.97 | 1.16 | 0.08 | G | CYT |  | Alpha-galactosidase |
| LA2921 | 6.92 | 17.07 | 1.09 | 0.09 | T | CYT |  | Receiver component of a two component response regulator |
| LA2924 | 4.05 | 15.37 | 0.84 | 0.08 | G | CYT | *pykF* | Pyruvate kinase |
| LA2930 | 4.01 | 18.51 | 0.43 | 0.03 | T | CYT |  | GGDEF domain protein |
| LA2935 | 3.47 | 27.62 | 0.86 | 0.09 | F | CYT | *purM* | Phosphoribosylformylglycinamidine cyclo-ligase |
| LA2936 | 2.00 | 17.17 | 2.41 | 0.35 | - | NON-CYT |  | LipL45-related lipoprotein |
| LA2947 | 34.63 | 78.37 | 0.95 | 0.03 | P | CYT | *sseA* | Thiosulfate sulfurtransferase |
| LA2950 | 2.24 | 21.80 | 0.93 | 0.09 | O | NON-CYT |  | Trypsin-like serine protease |
| LA2951 | 18.95 | 50.33 | 1.34 | 0.08 | T | CYT |  | Response regulator |
| LA2955 | 6.02 | 20.75 | 1.50 | 0.33 | I | CYT |  | Acyltransferase |
| LA2956 | 6.10 | 13.33 | 0.80 | 0.13 | I | CYT |  | Methylmalonyl-CoA mutase |
| LA2959 | 19.19 | 32.50 | 0.99 | 0.02 | E | CYT | *ilvD* | Dihydroxy-acid dehydratase |
| LA2966 | 8.00 | 23.53 | 0.88 | 0.16 | S | NON-CYT |  | Leucine-rich repeat protein |
| LA2967 | 4.00 | 23.08 | 1.14 | 0.13 | S | CYT |  | Conserved hypothetical protein |
| LA2970 | 4.00 | 33.00 | 1.14 | 0.14 | - | CYT |  | hypothetical protein |
| LA2973 | 11.79 | 56.51 | 0.76 | 0.05 | - | NON-CYT |  | Conserved hypothetical lipoprotein |
| LA2978 | 2.00 | 8.92 | 1.15 | 0.11 | - | IM |  | hypothetical lipoprotein |
| LA2980 | 17.30 | 35.83 | 0.36 | 0.02 | D | NON-CYT |  | Conserved hypothetical lipoprotein |
| LA2984 | 6.76 | 25.74 | 1.07 | 0.07 | - | CYT |  | Hypothetical protein |
| LA2988 | 16.09 | 57.22 | 1.26 | 0.02 | F | CYT | *pyrE* | Orotate phosphoribosyltransferase |
| LA2989 | 8.81 | 30.53 | 1.02 | 0.20 | H | CYT | *nadE* | NAD(+) synthase (glutamine-hydrolyzing) |
| LA2996 | 2.03 | 13.40 | 1.01 | 0.23 | C | CYT | *eutG* | Alcohol dehydrogenase, class IV |
| LA3005 | 2.00 | 21.74 | 1.49 | 0.17 | M | CYT |  | Conserved hypothetical protein |
| LA3028 | 10.00 | 20.15 | 0.22 | 0.01 | S | UNK |  | Leucine-rich repeat containing protein |
| LA3029 | 5.40 | 23.83 | 0.72 | 0.15 | R | CYT |  | Ankyrin repeat protein |
| LA3033 | 5.52 | 13.41 | 1.28 | 0.06 | S | CYT |  | Conserved hypothetical protein |
| LA3039 | 11.83 | 22.73 | 1.01 | 0.05 | - | NON-CYT |  | Hypothetical protein |
| LA3041 | 2.09 | 19.38 | 0.93 | 0.03 | T | CYT |  | Adenylyl- / guanylyl cyclase |
| LA3051 | 8.00 | 39.84 | 1.22 | 0.04 | M | CYT | *murI* | Glutamate racemase |
| LA3052 | 12.74 | 27.65 | 1.09 | 0.07 | E | CYT |  | Aspartate/tyrosine/aromatic aminotransferase |
| LA3053 | 2.00 | 13.04 | 1.11 | 0.02 | R | NON-CYT |  | Aminopeptidase |
| LA3067 | 14.79 | 19.24 | 0.78 | 0.04 | U | OM | *tolB* | Tol transport system component |
| LA3068 | 4.03 | 22.22 | 0.99 | 0.18 | - | NON-CYT |  | hypothetical lipoprotein |
| LA3069 | 11.52 | 30.14 | 0.87 | 0.09 | - | OM |  | hypothetical protein |
| LA3070 | 5.48 | 43.36 | 0.92 | 0.06 | T | CYT |  | Anti-Sigma factor antagonist |
| LA3080 | 12.72 | 30.48 | 1.09 | 0.06 | F | CYT | *purB* | Adenylosuccinate lyase |
| LA3084 | 20.25 | 33.22 | 1.23 | 0.05 | E | PER |  | Substrate binding protein of an ABC transporter complex |
| LA3086 | 8.36 | 22.70 | 0.77 | 0.04 | H | CYT | *nadC* | Nicotinate-nucleotide pyrophosphorylase |
| LA3091 | 12.18 | 42.97 | 0.98 | 0.11 | - | NON-CYT |  | hypothetical protein |
| LA3092 | 3.87 | 34.41 | 1.09 | 0.03 | - | CYT |  | hypothetical protein |
| LA3094 | 4.26 | 23.75 | 0.93 | 0.03 | P | CYT |  | FUR family transcriptional regulator |
| LA3095 | 2.00 | 11.23 | 1.39 | 0.22 | J | CYT | *tgt* | Queuine tRNA-ribosyltransferase |
| LA3096 | 12.32 | 73.87 | 1.07 | 0.03 | T | CYT |  | Anti-sigma factor antagonist |
| LA3097 | 2.02 | 19.28 | 0.82 | 0.02 | - | NON-CYT | *lipL71* | LipL71 lipoprotein |
| LA3100 | 2.28 | 9.83 | 1.20 | 0.20 | R | CYT |  | Hydrolase with alpha/beta fold domain |
| LA3104 | 6.03 | 28.62 | 2.18 | 0.15 | T | CYT |  | Signal transduction protein |
| LA3118 | 17.05 | 47.03 | 1.33 | 0.07 | - | NON-CYT |  | hypothetical lipoprotein |
| LA3119 | 2.00 | 3.06 | 0.89 | 0.09 | S | NON-CYT |  | Conserved hypothetical protein |
| LA3128 | 2.02 | 14.17 | 0.77 | 0.04 | R | CYT |  | Sulfurtransferase |
| LA3135 | 38.97 | 67.74 | 0.92 | 0.04 | I | CYT | *paaJ-2* | Acetyl-CoA acetyltransferase |
| LA3138 | 6.12 | 27.50 | 0.78 | 0.05 | - | OM | *ompL1* | OmpL1 |
| LA3143 | 20.32 | 43.15 | 0.84 | 0.03 | I | CYT |  | Acyl-CoA dehydrogenase |
| LA3144 | 4.02 | 13.08 | 0.78 | 0.06 | C | CYT | *caiB* | Acyl-CoA transferases/carnitine dehydratase |
| LA3147 | 20.66 | 56.32 | 0.81 | 0.03 | R | CYT |  | Alpha/beta hydrolase superfamily protein |
| LA3160 | 38.75 | 58.07 | 1.16 | 0.03 | I | CYT | *gcpE* | Deoxyxylulose biosynthesis protein |
| LA3170 | 6.00 | 22.79 | 0.78 | 0.09 | T | CYT |  | Serine phosphatase RsbU, regulator of sigma subunit |
| LA3172 | 4.00 | 35.97 | 1.12 | 0.11 | R | CYT |  | Aldo/keto reductase |
| LA3175 | 4.00 | 25.68 | 0.72 | 0.07 | - | CYT |  | hypothetical protein |
| LA3192 | 5.40 | 28.67 | 0.97 | 0.15 | - | CYT |  | Fruiting body developmental protein R-like protein |
| LA3214 | 5.09 | 32.34 | 2.18 | 0.31 | S | CYT |  | Conserved hypothetical protein |
| LA3216 | 2.03 | 20.69 | 1.22 | 0.06 | H | CYT | *ispA-2* | Geranyltranstransferase |
| LA3219 | 20.20 | 52.19 | 1.14 | 0.03 | G | CYT | *tktN* | Transketolase, N-terminal subunit |
| LA3239 | 4.00 | 25.00 | 1.24 | 0.21 | - | IM |  | Hypothetical protein |
| LA3240 | 30.98 | 48.81 | 0.85 | 0.01 | - | OM | *lipL48* | LipL48 lipoprotein |
| LA3241 | 2.00 | 24.70 | 0.89 | 0.11 | - | CYT |  | Hypothetical protein |
| LA3242 | 48.44 | 48.38 | 1.52 | 0.02 | P | OM |  | TonB-dependent receptor |
| LA3244 | 10.49 | 54.07 | 1.82 | 0.02 | U | IM | *tolQ* | Transport protein, TolQ-like |
| LA3267 | 14.45 | 22.61 | 0.85 | 0.02 | C | CYT |  | Fe-S-cluster-containing hydrogenase |
| LA3288 | 12.00 | 36.36 | 0.94 | 0.13 | E | CYT | *trpA* | Tryptophan synthase alpha chain |
| LA3290 | 27.36 | 43.58 | 0.92 | 0.05 | J | CYT | *proS* | Prolyl-tRNA ligase |
| LA3291 | 6.18 | 13.74 | 0.92 | 0.10 | M | IM |  | Zinc metalloprotease |
| LA3292 | 6.88 | 22.37 | 0.96 | 0.02 | I | CYT | *dxr* | 1-deoxy-D-xylulose-5-phosphate reductoisomerase |
| LA3297 | 11.15 | 38.69 | 1.01 | 0.05 | J | CYT | *tsf* | Translation elongation factor Ts |
| LA3298 | 43.53 | 86.73 | 0.71 | 0.01 | J | CYT | *rpsB* | 30S Ribosomal protein S2 |
| LA3306 | 12.31 | 32.99 | 1.30 | 0.06 | E | CYT | *dapA* | Dihydrodipicolinate synthase/N-acetylneuraminate lyase |
| LA3314 | 2.00 | 10.00 | 0.66 | 0.16 | R | CYT |  | Nucleoside-diphosphate-sugar epimerase |
| LA3316 | 6.12 | 15.25 | 0.42 | 0.01 | - | CYT |  | hypothetical protein |
| LA3342 | 4.02 | 15.74 | 1.28 | 0.11 | O | CYT |  | Protein-disulfide isomerase |
| LA3354 | 8.13 | 28.86 | 0.87 | 0.04 | R | CYT |  | Zn-dependent alcohol dehydrogenase |
| LA3356 | 2.59 | 17.86 | 2.24 | 0.09 | O | CYT | *gst-1* | glutathione transferase |
| LA3362 | 4.24 | 21.39 | 0.96 | 0.02 | R | CYT |  | Aminoglycoside phosphotransferase |
| LA3365 | 21.47 | 61.89 | 1.21 | 0.01 | H | CYT |  | Hydroxyethylthiazole kinase family |
| LA3366 | 2.00 | 15.32 | 0.23 | 0.06 | I | CYT | *caiD-1* | Enoyl-CoA hydratase/carnithine racemase |
| LA3368 | 2.08 | 40.54 | 0.90 | 0.10 | - | NON-CYT |  | hypothetical lipoprotein |
| LA3370 | 13.88 | 30.70 | 0.81 | 0.05 | S | OM |  | Surface Antigen OrfC lipoprotein |
| LA3372 | 6.78 | 28.57 | 1.14 | 0.12 | T | CYT |  | Cyclic nucleotide binding protein |
| LA3378 | 89.88 | 65.55 | 0.81 | 0.02 | S | CYT | *greA* | greA transcription elongation factor related protein |
| LA3379 | 11.31 | 39.37 | 0.30 | 0.02 | - | PER | *flaA-1* | Endoflagellar filament sheath protein |
| LA3380 | 2.00 | 12.55 | 0.33 | 0.01 | - | PER | *flaA-2* | Endoflagellar filament sheath protein |
| LA3399 | 4.14 | 24.36 | 1.08 | 0.30 | R | CYT |  | Metallo-beta-lactamase |
| LA3400 | 31.32 | 44.52 | 1.06 | 0.01 | R | UNK |  | Zn-dependent peptidase |
| LA3401 | 30.59 | 53.51 | 1.06 | 0.05 | R | NON-CYT |  | Zn-dependent peptidase |
| LA3403 | 4.00 | 33.33 | 1.31 | 0.04 | - | NON-CYT |  | Hypothetical lipoprotein |
| LA3407 | 35.97 | 34.60 | 1.01 | 0.05 | J | CYT | *alaS* | Alanine--tRNA ligase |
| LA3412 | 4.00 | 14.95 | 0.78 | 0.01 | H | CYT | *thiL* | Thiamine monophosphate kinase |
| LA3416 | 10.00 | 49.04 | 0.87 | 0.04 | J | CYT | *rpsG* | 30S Ribosomal protein S7 |
| LA3419 | 10.07 | 12.68 | 0.98 | 0.02 | K | CYT | *rpoC* | DNA-directed RNA polymerase, beta subunit |
| LA3421 | 16.15 | 82.68 | 0.68 | 0.04 | J | CYT | *rplL* | 50S Ribosomal protein L7/L12 |
| LA3422 | 10.92 | 48.59 | 0.84 | 0.09 | J | CYT | *rplJ* | 50S Ribosomal protein L10 |
| LA3423 | 11.34 | 40.43 | 0.94 | 0.05 | J | CYT | *rplA* | 50S Ribosomal protein L1 |
| LA3424 | 4.00 | 45.07 | 0.87 | 0.02 | J | CYT | *rplK* | 50S Ribosomal protein L11 |
| LA3425 | 15.03 | 69.06 | 1.26 | 0.06 | K | CYT | *nusG* | Transcription antiterminator |
| LA3432 | 7.43 | 13.14 | 0.84 | 0.06 | - | UNK |  | hypothetical protein |
| LA3435 | 2.00 | 9.44 | 0.88 | 0.19 | L | CYT |  | Superfamily II DNA and RNA helicase |
| LA3441 | 4.00 | 15.15 | 1.38 | 0.06 | E | CYT | *pepB* | Leucyl aminopeptidase |
| LA3453 | 2.00 | 3.77 | 0.72 | 0.18 | - | CYT |  | Conserved hypothetical protein |
| LA3461 | 12.00 | 42.80 | 1.15 | 0.15 | C | CYT |  | FAD/FMN-containing dehydrogenase |
| LA3468 | 45.27 | 44.95 | 1.06 | 0.02 | P | OM |  | Outer membrane receptor for Fe3+-dicitrate/TonB-dependent receptor |
| LA3469 | 12.46 | 24.09 | 0.37 | 0.01 | P | OM |  | Iron-regulated lipoprotein |
| LA3470 | 10.06 | 20.28 | 0.60 | 0.02 | C | NON-CYT |  | Thiol oxidoreductase |
| LA3471 | 12.00 | 38.37 | 1.01 | 0.08 | R | NON-CYT |  | Conserved hypothetical protein |
| LA3484 | 32.26 | 86.67 | 0.98 | 0.02 | Q | CYT |  | Short chain dehydrogenase |
| LA3485 | 7.79 | 25.53 | 1.42 | 0.11 | R | CYT |  | Esterase |
| LA3492 | 6.39 | 23.91 | 0.50 | 0.05 | O | NON-CYT |  | Protease |
| LA3497 | 4.61 | 12.43 | 1.54 | 0.27 | S | CYT |  | Conserved hypothetical protein |
| LA3511 | 10.03 | 49.77 | 1.29 | 0.30 | F | CYT |  | Phosphoribosylformylglycinamidine synthase, glutamine amidotransferase domain |
| LA3513 | 12.97 | 36.49 | 1.13 | 0.05 | F | CYT | *purC* | Phosphoribosylaminoimidazole-succinocarboxamide synthase |
| LA3514 | 2.04 | 16.98 | 0.76 | 0.06 | T | CYT |  | Serine phosphatase, regulator of sigma subunit |
| LA3560 | 9.41 | 30.99 | 1.38 | 0.12 | E | CYT | *csdB* | Selenocysteine lyase |
| LA3563 | 18.00 | 56.86 | 0.97 | 0.03 | O | CYT |  | ATP-binding component of an ABC transporter complex, Fe-S cluster assembly |
| LA3572 | 4.14 | 21.43 | 1.04 | 0.11 | T | CYT |  | Anti-sigma factor antagonist |
| LA3573 | 4.00 | 10.89 | 1.08 | 0.10 | M | CYT | *kdsB-1* | 3-deoxy-manno-octulosonate cytidylyltransferase |
| LA3575 | 8.57 | 44.25 | 1.07 | 0.02 | N | PER | *fliL* | Endoflagellar basal body-associated protein |
| LA3587 | 6.08 | 19.46 | 1.11 | 0.12 | R | CYT |  | Acetyltransferase |
| LA3597 | 14.30 | 33.69 | 0.84 | 0.07 | M | CYT |  | Pyridoxal-phosphate-dependent aminotransferase |
| LA3598 | 2.51 | 38.75 | 2.50 | 0.17 | P | CYT | *dps* | DNA-binding ferritin-like protein |
| LA3604 | 5.70 | 16.88 | 0.72 | 0.07 | I | CYT | *pldB-1* | Lysophospholipase |
| LA3606 | 8.28 | 56.00 | 0.85 | 0.08 | - | CYT |  | Conserved hypothetical protein |
| LA3607 | 7.00 | 38.41 | 0.87 | 0.05 | I | CYT |  | Acyl dehydratase, MaoC family |
| LA3608 | 10.00 | 37.81 | 0.84 | 0.10 | - | CYT |  | Conserved hypothetical protein |
| LA3615 | 2.11 | 6.36 | 0.65 | 0.05 | M | OM |  | OmpA-family protein |
| LA3625 | 24.87 | 31.41 | 0.71 | 0.02 | L | CYT | *polA* | DNA-directed DNA polymerase |
| LA3627 | 23.77 | 51.16 | 0.94 | 0.02 | I | CYT |  | Acyl-CoA dehydrogenase |
| LA3628 | 8.77 | 20.93 | 1.10 | 0.13 | E | CYT | *trpE* | Anthranilate synthase (component I) |
| LA3629 | 3.52 | 19.27 | 1.39 | 0.04 | E | CYT | *trpG* | Anthranilate synthase (component II) |
| LA3632 | 25.53 | 45.47 | 1.08 | 0.03 | C | NON-CYT |  | Conserved hypothetical protein |
| LA3636 | 5.52 | 14.39 | 0.93 | 0.27 | F | CYT |  | Cyclic amidohydrolase |
| LA3638 | 25.55 | 45.45 | 0.98 | 0.03 | E | CYT | *thrA* | Homoserine dehydrogenase |
| LA3639 | 4.49 | 44.35 | 0.92 | 0.01 | T | CYT |  | Anti-Sigma factor antagonist |
| LA3644 | 22.93 | 61.80 | 1.21 | 0.05 | I | CYT | *caiD-3* | Enoyl-CoA hydratase |
| LA3657 | 10.73 | 36.12 | 1.40 | 0.03 | T | CYT |  | Receiver component of a response regulator |
| LA3664 | 13.31 | 26.36 | 0.91 | 0.03 | - | NON-CYT |  | Conserved hypothetical protein |
| LA3665 | 11.00 | 50.00 | 0.87 | 0.04 | T | CYT |  | Anti-Sigma factor antagonist |
| LA3666 | 4.90 | 25.38 | 1.31 | 0.13 | F | CYT |  | Xanthosine triphosphate pyrophosphatase |
| LA3669 | 4.43 | 20.70 | 1.25 | 0.12 | - | NON-CYT |  | Hypothetical lipoprotein |
| LA3672 | 14.65 | 42.86 | 1.04 | 0.04 | I | NON-CYT |  | Hydrolase or acyltransferase, alpha/beta hydrolase superfamily, lipoprotein |
| LA3676 | 4.00 | 8.93 | 0.52 | 0.13 | I | CYT | *caiA-5* | Acyl-CoA dehydrogenase |
| LA3684 | 21.74 | 33.09 | 0.84 | 0.05 | R | IM |  | ATP-binding protein of an ABC transporter complex |
| LA3685 | 2.01 | 12.27 | 0.82 | 0.11 | M | OM |  | OmpA-family protein |
| LA3697 | 2.15 | 16.95 | 0.90 | 0.06 | S | NON-CYT |  | Conserved hypothetical protein |
| LA3704 | 6.88 | 37.74 | 1.93 | 0.11 | O | CYT | *grpE* | Chaperone protein, GrpE |
| LA3705 | 56.35 | 67.49 | 2.22 | 0.02 | O | CYT | *dnaK* | Chaperone protein, Hsp70 |
| LA3706 | 2.00 | 23.92 | 1.19 | 0.11 | O | CYT | *dnaJ* | Chaperone protein, DnaJ |
| LA3709 | 17.73 | 52.17 | 1.17 | 0.03 | R | CYT |  | Oxidoreductase |
| LA3710 | 12.00 | 54.40 | 1.39 | 0.13 | K | CYT |  | Transcriptional regulator |
| LA3714 | 34.09 | 32.33 | 1.04 | 0.03 | J | CYT | *leuS* | Leucine--tRNA ligase |
| LA3722 | 11.66 | 34.19 | 1.00 | 0.01 | - | NON-CYT |  | hypothetical lipoprotein |
| LA3724 | 6.85 | 32.67 | 0.86 | 0.05 | - | NON-CYT |  | hypothetical lipoprotein |
| LA3737 | 2.09 | 18.31 | 0.98 | 0.04 | M | IM | *hlyD* | Heavy metal efflux pump |
| LA3744 | 8.00 | 22.97 | 1.24 | 0.21 | - | CYT |  | hypothetical protein |
| LA3747 | 35.47 | 64.37 | 1.20 | 0.02 | E | CYT | *thrC* | Threonine synthase |
| LA3748 | 19.13 | 43.33 | 1.42 | 0.07 | S | CYT |  | Conserved hypothetical protein |
| LA3749 | 4.00 | 25.91 | 1.01 | 0.09 | - | CYT |  | hypothetical protein |
| LA3758 | 6.00 | 48.84 | 0.14 | 0.02 | R | CYT |  | RNA-binding protein |
| LA3762 | 3.22 | 9.15 | 0.79 | 0.06 | - | IM |  | hypothetical protein |
| LA3763 | 38.85 | 36.96 | 0.99 | 0.03 | J | CYT | *valS* | Valine--tRNA ligase |
| LA3764 | 14.28 | 30.52 | 1.08 | 0.03 | F | CYT | *purD* | Phosphoribosylamine--glycine ligase |
| LA3767 | 11.52 | 28.07 | 0.91 | 0.08 | J | CYT | *prfB* | Peptide chain release factor 2 |
| LA3769 | 2.00 | 28.39 | 0.24 | 0.01 | - | CYT |  | hypothetical protein |
| LA3783 | 5.40 | 17.42 | 0.85 | 0.04 | F | CYT | *add* | Adenosine deaminase |
| LA3788 | 6.00 | 27.56 | 1.18 | 0.11 | R | CYT |  | Hydrolase or Acyltransferase |
| LA3793 | 6.00 | 23.36 | 1.59 | 0.16 | R | CYT |  | Conserved hypothetical protein |
| LA3795 | 4.00 | 17.32 | 0.99 | 0.08 | M | CYT |  | Glycosyltransferase |
| LA3796 | 2.00 | 17.49 | 1.03 | 0.27 | - | CYT |  | Conserved hypothetical protein |
| LA3800 | 14.00 | 30.22 | 1.16 | 0.05 | G | CYT | *manB-1* | Phosphomannomutase |
| LA3801 | 18.30 | 23.44 | 0.90 | 0.03 | M | CYT | *glmS* | Glucosamine 6-phosphate synthetase |
| LA3807 | 4.44 | 39.47 | 3.70 | 0.42 | E | CYT | *glnK* | Nitrogen regulatory protein PII |
| LA3814 | 2.00 | 5.99 | 0.22 | 0.01 | - | CYT |  | Conserved hypothetical protein |
| LA3820 | 10.72 | 52.41 | 1.00 | 0.02 | J | CYT | *pth-2* | Aminoacyl-tRNA hydrolase |
| LA3821 | 10.82 | 57.55 | 0.98 | 0.03 | J | CYT | *rplY* | 50S Ribosomal protein L25 |
| LA3822 | 4.00 | 14.74 | 0.75 | 0.05 | F | CYT | *prsA* | Ribose-phosphate diphosphokinase |
| LA3823 | 6.00 | 23.81 | 0.97 | 0.23 | M | CYT | *glmU* | UDP-N-acetylglucosamine diphosphorylase |
| LA3825 | 8.46 | 28.34 | 0.99 | 0.04 | K | CYT |  | Transcriptional regulator |
| LA3833 | 2.00 | 2.37 | 0.76 | 0.24 | - | CYT |  | Conserved hypothetical protein |
| LA3838 | 6.00 | 20.18 | 0.90 | 0.11 | - | NON-CYT |  | Conserved hypothetical protein |
| LA3845 | 3.85 | 20.00 | 1.12 | 0.22 | K | CYT |  | Transcriptional regulator, AcrR-family |
| LA3861 | 10.32 | 38.60 | 1.28 | 0.09 | - | CYT |  | hypothetical protein |
| LA3870 | 6.00 | 17.33 | 0.97 | 0.22 | - | NON-CYT |  | hypothetical protein |
| LA3874 | 15.15 | 58.80 | 1.44 | 0.05 | R | CYT | *surE* | Acid phosphatase |
| LA3879 | 5.40 | 21.05 | 1.15 | 0.17 | R | CYT |  | Nucleoside-diphosphate sugar epimerase |
| LA3884 | 2.00 | 6.80 | 1.01 | 0.19 | T | CYT |  | Anti-sigma factor antagonist |
| LA3888 | 4.14 | 24.49 | 1.29 | 0.26 | G | CYT | *pgi* | Glucose-6-phosphate isomerase |
| LA3892 | 2.03 | 18.60 | 0.93 | 0.06 | M | CYT |  | Nucleoside-diphosphate-sugar pyrophosphorylase |
| LA3893 | 3.78 | 18.73 | 0.92 | 0.09 | R | CYT |  | Serine/Threonine protein kinase |
| LA3917 | 4.00 | 21.89 | 1.45 | 0.08 | - | CYT |  | Hypothetical protein |
| LA3918 | 33.56 | 38.18 | 1.15 | 0.04 | J | CYT | *metG* | Methionine--tRNA ligase |
| LA3924 | 4.00 | 19.00 | 0.97 | 0.24 | M | CYT |  | 3-beta hydroxysteroid dehydrogenase/isomerase family |
| LA3928 | 12.15 | 28.50 | 0.93 | 0.10 | I | CYT | *caiA-1* | Acyl-CoA dehydrogenase |
| LA3929 | 6.00 | 25.69 | 0.70 | 0.02 | T | CYT |  | Response regulator with GGDEF domain |
| LA3930 | 2.31 | 12.89 | 0.78 | 0.18 | M | CYT | *murB* | UDP-N-acetylmuramate dehydrogenase |
| LA3935 | 4.77 | 14.91 | 1.10 | 0.10 | H | CYT | *coaB* | Phosphopantothenate--cysteine ligase |
| LA3938 | 12.82 | 18.35 | 0.93 | 0.16 | - | NON-CYT |  | Hypothetical protein |
| LA3939 | 8.36 | 30.45 | 0.97 | 0.12 | C | CYT | *gpsA* | Glycerol-3-phosphate dehydrogenase (NAD(P)+) |
| LA3941 | 4.00 | 17.42 | 1.32 | 0.27 | R | CYT |  | Phosphoesterase |
| LA3948 | 15.94 | 49.54 | 1.11 | 0.02 | M | CYT | *galE* | UDP-glucose 4-epimerase |
| LA3957 | 23.25 | 56.57 | 1.08 | 0.01 | E | CYT | *aroF* | 3-deoxy-D-arabino-heptulosonate 7-phosphate (DAHP) synthase |
| LA3959 | 8.08 | 48.99 | 1.50 | 0.16 | H | CYT | *folK* | 2-amino-4-hydroxy-6-hydroxymethyldihydropteridine pyrophosphokinase |
| LA3960 | 2.39 | 5.26 | 1.39 | 0.09 | L | CYT | *dnaC* | DNA replication protein |
| LA3961 | 6.00 | 19.28 | 0.52 | 0.05 | - | NON-CYT |  | hypothetical protein |
| LA3964 | 21.57 | 44.12 | 0.87 | 0.04 | M | CYT | *gmd* | GDP-mannose 4,6-dehydratase |
| LA3969 | 10.00 | 27.44 | 1.13 | 0.03 | F | CYT |  | Adenosine deaminase |
| LA3977 | 4.10 | 27.16 | 1.37 | 0.04 | L | CYT | *nudH* | NTP pyrophosphohydrolase |
| LA3982 | 4.00 | 26.77 | 0.74 | 0.13 | - | CYT |  | Conserved hypothetical protein |
| LA3998 | 6.00 | 13.39 | 0.80 | 0.08 | E | CYT | *betA* | Choline dehydrogenase |
| LA4016 | 2.00 | 7.14 | 0.49 | 0.08 | Q | CYT |  | Conserved hypothetical protein |
| LA4035 | 14.66 | 22.52 | 0.94 | 0.06 | J | CYT | *pheT* | Phenylalanine--tRNA ligase beta subunit |
| LA4040 | 2.00 | 12.36 | 0.72 | 0.09 | C | CYT |  | Inorganic pyrophosphatase |
| LA4052 | 6.07 | 28.20 | 1.16 | 0.03 | R | CYT |  | Short chain dehydrogenase |
| LA4056 | 8.00 | 34.62 | 0.88 | 0.14 | R | CYT |  | Ankyrin-repeat protein |
| LA4058 | 6.00 | 28.65 | 0.95 | 0.05 | R | CYT |  | Lysine decarboxylase-related protein |
| LA4063 | 2.00 | 12.08 | 1.72 | 0.19 | R | CYT |  | Pyrophosphatase |
| LA4066 | 4.00 | 14.84 | 0.69 | 0.13 | - | NON-CYT |  | hypothetical protein |
| LA4067 | 45.39 | 64.57 | 1.02 | 0.03 | C | CYT | *icdA* | Isocitrate dehydrogenase |
| LA4068 | 4.10 | 20.42 | 1.06 | 0.09 | - | CYT |  | Hypothetical protein |
| LA4084 | 4.25 | 32.24 | 0.87 | 0.06 | - | NON-CYT |  | Hypothetical lipoprotein |
| LA4089 | 7.15 | 29.93 | 1.24 | 0.09 | R | CYT |  | PhzC/PhzF-related epimerase |
| LA4091 | 2.28 | 10.61 | 1.11 | 0.23 | J | CYT | *rnd* | Ribonuclease III |
| LA4092 | 4.00 | 12.37 | 0.65 | 0.10 | F | CYT | *purF* | Amidophosphoribosyltransferase |
| LA4093 | 2.00 | 21.08 | 1.09 | 0.15 | L | CYT |  | NUDIX domain protein |
| LA4102 | 10.35 | 52.36 | 0.95 | 0.04 | T | CYT |  | Response regulator |
| LA4109 | 9.70 | 27.27 | 0.99 | 0.12 | - | CYT |  | Conserved hypothetical protein |
| LA4110 | 2.12 | 16.67 | 0.97 | 0.05 | - | CYT |  | hypothetical protein |
| LA4119 | 15.19 | 29.37 | 1.23 | 0.07 | L | CYT | *lig* | DNA ligase (NAD+) (contains BRCT domain type II) |
| LA4131 | 4.19 | 18.36 | 1.20 | 0.05 | R | NON-CYT |  | Zinc dependent protease |
| LA4138 | 32.87 | 53.21 | 1.23 | 0.04 | I | CYT | *fadB* | 3-hydroxyacyl-CoA dehydrogenase |
| LA4139 | 14.74 | 27.55 | 1.38 | 0.03 | I | CYT | *paaJ-5* | Acetyl-CoA acetyltransferase |
| LA4150 | 3.70 | 9.09 | 1.12 | 0.06 | J | CYT | *pheS* | Phenylalanine-tRNA synthetase alpha subunit |
| LA4151 | 4.27 | 18.28 | 0.99 | 0.09 | M | NON-CYT | *murC-2* | UDP-N-acetylmuramate--L-alanine ligase |
| LA4153 | 6.22 | 21.25 | 1.22 | 0.08 | - | NON-CYT |  | hypothetical protein |
| LA4154 | 9.22 | 15.33 | 1.18 | 0.13 | - | IM |  | Conserved hypothetical protein |
| LA4156 | 12.47 | 39.81 | 0.96 | 0.10 | V | CYT |  | ATP-binding protein of an ABC transporter complex |
| LA4159 | 31.64 | 63.12 | 1.09 | 0.04 | I | CYT | *fabD* | [Acyl-carrier protein] S-malonyltransferase |
| LA4165 | 24.22 | 53.85 | 0.99 | 0.01 | E | CYT | *argG* | Argininosuccinate synthase |
| LA4167 | 7.11 | 38.75 | 1.13 | 0.02 | H | CYT | *coaD* | Pantetheine-phosphate adenylyltransferase |
| LA4168 | 15.89 | 70.07 | 1.13 | 0.02 | F | CYT | *ndk* | Nucleoside-diphosphate kinase |
| LA4171 | 25.68 | 36.07 | 1.17 | 0.01 | E | CYT | *leuB-2* | Isocitrate dehydrogenase (NADP+) |
| LA4174 | 4.00 | 10.60 | 0.74 | 0.05 | T | CYT |  | Sensor histidine kinase of a two component response regulator |
| LA4185 | 2.00 | 8.80 | 1.55 | 0.48 | - | CYT |  | hypothetical protein |
| LA4190 | 36.14 | 51.06 | 1.08 | 0.02 | J | CYT | *glnS* | Bifunctional glutamate--tRNA ligase/glutamine--tRNA ligase |
| LA4193 | 10.31 | 21.86 | 1.18 | 0.15 | L | CYT |  | DNA topoisomerase (ATP-hydrolyzing), subunit A |
| LA4194 | 2.46 | 13.92 | 0.88 | 0.02 | L | CYT |  | DNA topoisomerase (ATP-hydrolyzing), subunit B |
| LA4198 | 10.59 | 27.86 | 0.26 | 0.01 | - | CYT |  | hypothetical protein |
| LA4200 | 6.00 | 21.48 | 0.91 | 0.11 | E | IM | *aofA* | Amine oxidase (flavin-containing) |
| LA4208 | 10.00 | 27.00 | 0.41 | 0.07 | - | NON-CYT |  | Conserved hypothetical protein |
| LA4209 | 8.62 | 18.13 | 0.47 | 0.05 | - | NON-CYT |  | Conserved hypothetical protein |
| LA4212 | 2.00 | 9.30 | 1.47 | 0.21 | - | NON-CYT |  | Hypothetical protein |
| LA4216 | 46.01 | 56.68 | 1.01 | 0.01 | P | CYT | *cysI* | Sulfite reductase (NADPH), alpha subunit |
| LA4219 | 14.86 | 34.38 | 1.12 | 0.07 | P | CYT | *cysN* | Sulfate adenylyltransferase |
| LA4220 | 8.51 | 31.15 | 1.07 | 0.01 | H | CYT | *cysH-2* | Phosphoadenylyl-sulfate reductase (thioredoxin) |
| LA4221 | 8.64 | 28.86 | 1.36 | 0.08 | H | CYT | *cysH-1* | Phosphoadenylyl-sulfate reductase (thioredoxin) |
| LA4224 | 10.00 | 18.36 | 0.40 | 0.02 | C | NON-CYT |  | FAD-dependent oxidoreductase |
| LA4231 | 2.13 | 19.87 | 0.96 | 0.18 | E | CYT |  | ATP-binding protein of an ABC transporter complex |
| LA4240 | 25.53 | 34.63 | 0.71 | 0.01 | - | NON-CYT |  | Conserved hypothetical protein |
| LA4242 | 37.26 | 66.37 | 0.84 | 0.03 | H | CYT | *ilvC* | Ketol-acid reductoisomerase |
| LA4246 | 2.03 | 7.22 | 0.70 | 0.11 | P | PER | *phoD* | Phosphodiesterase I |
| LA4248 | 17.70 | 36.93 | 1.01 | 0.03 | F | CYT |  | Purine-nucleoside phosphorylase |
| LA4266 | 19.48 | 43.41 | 0.66 | 0.08 | F | CYT | *purT* | Phosphoribosylglycinamide formyltransferase |
| LA4270 | 2.00 | 8.39 | 0.99 | 0.27 | R | IM |  | Permease |
| LA4272 | 4.68 | 15.08 | 0.99 | 0.22 | - | NON-CYT |  | Hypothetical protein |
| LA4277 | 19.72 | 34.98 | 0.91 | 0.04 | C | CYT | *hycE* | Metal (Ni/Fe) hydrogenase, large subunit |
| LA4290 | 6.28 | 15.97 | 0.91 | 0.02 | F | CYT | *pyrD* | Dihydroorotate dehydrogenase |
| LA4291 | 12.00 | 29.17 | 0.76 | 0.09 | - | NON-CYT |  | hypothetical lipoprotein |
| LA4300 | 4.00 | 18.50 | 0.49 | 0.08 | R | CYT |  | ThiJ/PfpI family intracellular protease |
| LA4307 | 6.00 | 32.67 | 1.29 | 0.21 | S | CYT |  | Conserved hypothetical protein |
| LA4318 | 2.00 | 16.94 | 0.92 | 0.09 | - | NON-CYT |  | Hypothetical lipoprotein |
| LA4322 | 16.60 | 29.57 | 1.13 | 0.04 | E | OM |  | Zn-dependent carboxypeptidase |
| LA4326 | 6.55 | 22.44 | 0.48 | 0.07 | M | CYT | *lpxD-1* | UDP-3-O-[3-hydroxymyristoyl] glucosamine N-acyltransferase |
| LA4327 | 9.70 | 21.29 | 1.14 | 0.04 | P | CYT |  | Reductase |
| LA4329 | 2.00 | 18.31 | 1.52 | 0.05 | - | CYT |  | Hypothetical protein |
| LA4333 | 2.00 | 13.71 | 0.73 | 0.08 | L | CYT | *recR* | Recombinational DNA repair protein |
| LA4336 | 5.52 | 16.67 | 1.37 | 0.05 | E | PER |  | Substrate binding protein of an ABC transporter complex |
| LA4339 | 16.47 | 39.09 | 1.00 | 0.03 | J | CYT | *serS* | Serine--tRNA ligase |
| LA4340 | 5.24 | 28.62 | 1.14 | 0.21 | L | CYT | *tatD* | Mg-dependent DNase |
| LA4346 | 9.40 | 26.12 | 1.03 | 0.03 | K | CYT |  | ParB-like protein |
| LA4359 | 4.00 | 7.72 | 0.85 | 0.26 | D | CYT | *gidA* | Glucose inhibited division protein A |
| LA4360 | 10.15 | 30.62 | 1.05 | 0.07 | E | CYT | *ilvE* | Branched-chain amino acid aminotransferase |
| LB001 | 4.05 | 15.39 | 0.83 | 0.10 | - | OM |  | Conserved hypothetical protein |
| LB002 | 10.09 | 34.47 | 0.97 | 0.04 | E | CYT | *metF* | 5,10-methylenetetrahydrofolate reductase |
| LB005 | 15.41 | 46.35 | 1.03 | 0.06 | R | OM |  | Conserved hypothetical protein |
| LB011 | 18.26 | 36.72 | 0.87 | 0.08 | H | CYT | *hemCD* | Bifunctional porphobilinogen deaminase/uroporphyrinogen synthase |
| LB013 | 24.50 | 53.50 | 1.13 | 0.07 | H | CYT | *hemL* | Glutamate-1-semialdehyde aminotransferase |
| LB015 | 6.22 | 18.26 | 1.02 | 0.06 | T | CYT |  | Response regulator of a two component regulatory system |
| LB016 | 8.85 | 30.59 | 0.93 | 0.02 | H | CYT | *hemE* | Uroporphyrinogen decarboxylase |
| LB017 | 12.84 | 29.84 | 0.99 | 0.04 | H | CYT | *hemN* | Coproporphyrinogen oxidase |
| LB026 | 5.30 | 12.13 | 0.87 | 0.09 | D | CYT |  | ParA-like protein |
| LB027 | 2.00 | 4.26 | 1.57 | 0.34 | K | CYT |  | ParB-like protein |
| LB035 | 6.00 | 24.59 | 2.14 | 0.13 | T | CYT | *wzb* | Protein-tyrosine-phosphatase |
| LB039 | 4.00 | 10.40 | 1.41 | 0.24 | - | NON-CYT |  | hypothetical protein |
| LB042 | 10.00 | 47.86 | 1.21 | 0.20 | S | CYT |  | Conserved hypothetical protein |
| LB047 | 19.02 | 30.75 | 1.03 | 0.02 | S | NON-CYT |  | Conserved hypothetical lipoprotein |
| LB048 | 2.00 | 15.13 | 1.21 | 0.21 | L | CYT |  | Transposase, ISlin1 |
| LB051 | 21.48 | 50.15 | 1.45 | 0.06 | R | CYT |  | MoxR-like ATPase |
| LB058 | 57.20 | 71.33 | 1.27 | 0.03 | O | CYT | *htpG* | HSP90 molecular chaperone |
| LB062 | 3.70 | 22.81 | 1.08 | 0.18 | T | CYT |  | Antagonist of anti-sigma factor |
| LB074 | 45.09 | 51.94 | 1.03 | 0.03 | I | CYT | *meaA* | Methylmalonyl-CoA mutase |
| LB079 | 4.01 | 41.30 | 1.70 | 0.24 | E | CYT |  | Phosphoribosyl-ATP diphosphatase |
| LB082 | 24.46 | 68.90 | 0.89 | 0.02 | R | CYT |  | Short chain dehydrogenase |
| LB083 | 9.80 | 62.34 | 0.99 | 0.03 | I | CYT | *acpP* | Acyl carrier protein |
| LB090 | 2.00 | 7.82 | 0.99 | 0.11 | - | CYT |  | Conserved hypothetical protein |
| LB098 | 13.60 | 26.05 | 0.90 | 0.07 | G | NON-CYT |  | polysaccharide deacetylase |
| LB105 | 6.00 | 15.34 | 0.88 | 0.06 | H | CYT |  | Methylase/methyltransferase |
| LB106 | 33.49 | 55.73 | 0.86 | 0.03 | H | CYT | *sahH* | S-adenosylhomocysteine hydrolase |
| LB108 | 53.35 | 39.21 | 0.76 | 0.02 | E | CYT | *metH* | Methionine synthase |
| LB112 | 5.40 | 13.43 | 0.68 | 0.13 | T | CYT |  | Serine phosphatase RsbU, regulator of sigma subunit |
| LB113 | 12.33 | 47.42 | 1.20 | 0.02 | Q | CYT |  | Short chain dehydrogenase |
| LB114 | 4.00 | 13.55 | 1.39 | 0.21 | E | CYT | *argB* | Acetylglutamate kinase |
| LB124 | 15.17 | 45.58 | 1.33 | 0.08 | T | CYT |  | Signal transduction protein |
| LB125 | 8.01 | 58.33 | 1.19 | 0.08 | N | CYT |  | Chemotaxis protein |
| LB127 | 4.00 | 20.32 | 1.23 | 0.06 | - | NON-CYT |  | hypothetical lipoprotein |
| LB136 | 8.35 | 63.64 | 0.57 | 0.03 | T | CYT |  | Anti-sigma factor antagonist |
| LB143 | 3.46 | 15.98 | 1.32 | 0.04 | S | CYT |  | LipL45-related protein |
| LB169 | 5.87 | 34.42 | 1.40 | 0.07 | - | CYT |  | Hypothetical protein |
| LB176 | 12.77 | 55.93 | 1.10 | 0.09 | F | CYT |  | Phosphoribosyltransferase |
| LB177 | 24.00 | 33.33 | 0.93 | 0.02 | O | NON-CYT |  | Trypsin-like serine protease |
| LB178 | 20.35 | 37.00 | 0.94 | 0.03 | O | NON-CYT |  | Trypsin-like serine protease |
| LB186 | 13.22 | 29.33 | 1.62 | 0.11 | P | CYT |  | Heme oxygenase |
| LB191 | 16.32 | 32.47 | 1.26 | 0.14 | H | OM |  | TonB-dependant outer membrane receptor |
| LB192 | 6.03 | 30.04 | 1.02 | 0.08 | - | NON-CYT |  | Hypothetical lipoprotein |
| LB194 | 14.26 | 66.15 | 0.71 | 0.03 | - | NON-CYT |  | Hypothetical lipoprotein |
| LB198 | 2.00 | 24.75 | 1.65 | 0.45 | S | CYT |  | conserved hypothetical protein |
| LB204 | 4.00 | 14.47 | 1.20 | 0.28 | M | CYT |  | Glycosyltransferase |
| LB209 | 4.00 | 16.58 | 0.81 | 0.09 | - | CYT |  | Hypothetical protein |
| LB211 | 2.00 | 26.20 | 0.93 | 0.15 | - | NON-CYT |  | Hypothetical protein |
| LB222 | 4.10 | 54.67 | 1.36 | 0.18 | T | CYT | *dksA* | DnaK suppressor protein |
| LB242 | 9.39 | 32.62 | 2.01 | 0.19 | - | NON-CYT |  | LipL45-related lipoprotein |
| LB248 | 7.62 | 23.51 | 0.57 | 0.04 | - | NON-CYT |  | Conserved hypothetical protein |
| LB250 | 4.00 | 17.20 | 0.86 | 0.05 | - | NON-CYT |  | Hypothetical lipoprotein |
| LB258 | 21.18 | 28.79 | 1.06 | 0.03 | O | NON-CYT |  | Cysteine protease |
| LB267 | 12.00 | 24.02 | 1.03 | 0.05 | E | CYT | *csdB* | Selenocysteine lyase |
| LB268 | 6.00 | 15.22 | 0.81 | 0.04 | S | CYT |  | Conserved hypothetical protein |
| LB273 | 35.28 | 50.80 | 1.08 | 0.01 | I | CYT | *sbm* | Methylmalonyl-COA mutase |
| LB274 | 34.33 | 41.26 | 1.15 | 0.03 | I | CYT |  | Methylmalonyl-CoA mutase |
| LB276 | 5.70 | 56.00 | 0.91 | 0.13 | - | NON-CYT |  | Hypothetical protein |
| LB277 | 2.00 | 5.24 | 0.88 | 0.23 | - | NON-CYT |  | Hypothetical protein |
| LB278 | 2.00 | 7.38 | 0.93 | 0.03 | - | CYT |  | Hypothetical protein |
| LB279 | 12.50 | 21.67 | 0.92 | 0.04 | P | OM |  | TonB dependent receptor |
| LB288 | 2.00 | 7.53 | 0.75 | 0.22 | R | CYT |  | Thioesterase |
| LB289 | 2.00 | 24.35 | 0.42 | 0.13 | - | CYT |  | Hypothetical protein |
| LB293 | 5.35 | 57.63 | 0.59 | 0.05 | S | CYT |  | ARD family protein |
| LB294 | 11.05 | 40.96 | 0.87 | 0.02 | J | CYT |  | Translation elongation factor P |
| LB298 | 2.06 | 7.95 | 1.39 | 0.05 | E | CYT | *kamA* | Lysine 2,3-aminomutase |
| LB299 | 2.20 | 28.23 | 0.70 | 0.06 | - | CYT |  | Hypothetical protein |
| LB310 | 7.81 | 28.41 | 1.40 | 0.06 | F | CYT | *pyrF* | Orotidine-5'-phosphate decarboxylase |
| LB311 | 14.34 | 37.50 | 1.08 | 0.01 | E | CYT | *speE* | Spermidine synthase |
| LB316 | 8.00 | 32.10 | 0.88 | 0.04 | - | CYT |  | Hypothetical protein |
| LB327 | 60.67 | 58.92 | 0.91 | 0.01 | C | CYT | *acnA* | Aconitate hydratase |
| LB329 | 2.00 | 3.40 | 0.88 | 0.20 | J | CYT |  | tRNA synthetase class II (D, K and N) |
| LB333 | 22.13 | 58.09 | 0.76 | 0.04 | T | CYT |  | Response regulator |
| LB335 | 4.00 | 29.90 | 1.54 | 0.22 | H | CYT | *phhB* | 4a-hydroxytetrahydrobiopterin dehydratase |
| LB353 | 14.11 | 30.86 | 0.97 | 0.18 | G | CYT | *pykF* | Pyruvate kinase |
| LB354 | 10.19 | 16.69 | 1.30 | 0.08 | - | CYT |  | Hypothetical protein |
| LB355 | 22.25 | 53.01 | 1.06 | 0.05 | E | CYT | *asd* | Aspartate-semialdehyde dehydrogenase |
| LB361 | 4.00 | 32.81 | 0.97 | 0.05 | - | CYT |  | hypothetical protein |
| LB363 | 14.05 | 38.69 | 0.87 | 0.03 | - | CYT |  | Hypothetical protein |
| LB365 | 14.15 | 46.40 | 1.04 | 0.12 | D | CYT |  | ParA-like protein |
| LB366 | 14.02 | 40.93 | 0.89 | 0.05 | K | CYT |  | ParB-like protein |

*^a^*Score is a measure of the confidence in the assignment. An assignment at 99% yields a score of 2.

*^b^*Percentage of the protein sequence that was identified by MS/MS peptide assignments.

*^c^*Mean fold change of protein abundance in overnight 37°C upshift cultures compared to 30°C cultures.

*^d^*COG categories and predicted locations as in Table 2.
